# Fertility Preservation and Infertility Treatment in Medical Training: An Assessment of Residency and Fellowship Program Directors' Attitudes

**DOI:** 10.1089/whr.2021.0044

**Published:** 2021-12-07

**Authors:** Megan Huynh, Ange Wang, Jacqueline Ho, Christopher N. Herndon, Lusine Aghajanova

**Affiliations:** ^1^ School of Nursing and Health Studies, Georgetown University, Washington, District of Columbia, USA.; ^2^ Division of Reproductive Endocrinology and Infertility, Department of Obstetrics, Gynecology and Reproductive Sciences, University of California, San Francisco, San Francisco, California, USA.; ^3^ Division of Reproductive Endocrinology and Infertility, Department of Obstetrics & Gynecology, USC Keck School of Medicine, Los Angeles, California, USA.; ^4^ Division of Reproductive Endocrinology and Infertility, Department of Obstetrics and Gynecology, University of Washington School of Medicine, Seattle, Washington, USA.; ^5^ Division of Reproductive Endocrinology and Infertility, Department of Obstetrics and Gynecology, Stanford University School of Medicine, Sunnyvale, California, USA.

**Keywords:** family planning, fellowship, fertility preservation, infertility, program directors, residency

## Abstract

***Background:*** Given the concurrence of medical residency and fellowship training with typical childbearing years, trainees often must make difficult decisions regarding family planning, requiring the support of their residency and fellowship program directors (PDs) to guide them.

***Objective:*** Our hypothesis was that PDs have knowledge gaps and varying levels of support in terms of their trainees' fertility, and the goal of our study was to assess the knowledge and support of residency and fellowship PDs in the United States toward trainees' reproductive needs.

***Methods:*** Cross-sectional survey distributed to all residency and fellowship PDs providing contact information through the Accreditation Council for Graduate Medical Education website in August 2019.

***Results:*** Of 299 respondents, the most common lengths of leave reported were 6–8 weeks of maternity leave and under 2 weeks of paternity leave. A total of 57.2% did not know their program's insurance for infertility treatment, and 68.6% did not know fertility preservation coverage. A total of 52.2% of PDs were unaware of if their trainees faced infertility. PDs supported residents' needs through moral support (68.2%) and time off for appointments (65.2%). Similarly, most PDs (66.2%) never had a trainee express interest in fertility preservation to them but offered moral support (59.2%) and time off (48.5%). Respondents felt it was important to increase resources for trainees by increasing their awareness of needs (47.5%) and establishing reproduction-related policies (34.1%).

***Conclusion:*** The study found variations regarding PDs' knowledge and support levels for trainees' fertility needs. Most were unaware of their trainees' fertility needs, and many PDs felt it would be important to improve resources by increasing personal awareness and creating policies for support to promote reproductive health equity for trainees.

## Introduction

The ability to reproduce lasts impermanently, and age is most correlated with fertility, which can affect decision making during this crucial time.^1,2^ On average, women face a slight decline in fertility around age 25–29, with a more drastic decrease at age 35–39, while data suggest that male fecundity declines around age 45.^3–5^ Infertility is the inability to conceive after 1 year of regular unprotected sexual intercourse for women younger than 35 years and after 6 months for women older than 35 years.^6,7^ In the United States, infertility among women aged 15–44 has a prevalence of 15.5%, which increases with age, while the prevalence of male infertility is 8%–12%.^8,9^

Recurrent pregnancy loss (RPL) is the loss of two consecutive pregnancies before 20 weeks from the last menstrual period, and its prevalence is 1%–2%.^2,7,10,11^ Increasing maternal age may be an underlying risk factor for RPL and other pregnancy complications such as preterm birth and stillbirths.^12,13^

Because medical training typically occurs during prime reproductive years, physicians-in-training are often faced with difficult family planning decisions. In fact, a study of female physicians in the United States found that nearly one-quarter reported being diagnosed with infertility, a higher prevalence than in the general population.^14^ Given the high-stress and time-consuming workload of training, residents and fellows often choose to delay having children.^15–20^ In addition, because of age-related fertility decline, medical trainees may need to undergo infertility treatment or desire fertility preservation. Oocyte cryopreservation has become widely utilized since 2013, when the American Society of Reproductive Medicine removed the technique's designation as “experimental.”^21–24^

Postgraduate medical education can delay childbearing by years. While trainees have demonstrated interest in fertility preservation, lack of support from residency and fellowship program directors (PDs) discourages them from pursuing treatment.^14,25^ Examining PDs' attitudes toward infertility treatment and fertility preservation is important to increase the understanding and support for trainees. Literature is limited in this area, and a lack of awareness may contribute to reproductive inequities. Opinions of PDs may be critical to improving support for residents to access fertility treatment. Our study is the first to our knowledge to examine residency and fellowship PDs in the United States across specialties to assess their knowledge and support regarding infertility treatment and fertility cryopreservation for their trainees.

## Methods

This study was a cross-sectional survey conducted through Qualtrics, an online survey software. The survey was distributed to all residency and fellowship PDs providing contact information through the Accreditation Council for Graduate Medical Education (ACGME) website from May and June 2019. The study received Institutional Review Board approval from Stanford University.

The survey was sent out to e-mail addresses of programs on the ACGME website, which were manually extracted from the site. Primarily, the e-mail address listed was for the PDs themselves, but if the program coordinators' contact was listed, they were asked to forward the survey to the PD. Programs were sent one reminder to increase response rates. While the survey was sent to 4366 programs, it is uncertain how many PDs actually received the e-mail, as some e-mails bounced, and it was not possible to determine if program coordinators forwarded the e-mail to PDs. Therefore, the final response rate cannot be determined due to indirect distribution and no confirmation on the number of people receiving the survey.

The questions asked in this survey were based on a previously conducted pilot survey assessing the fertility and reproductive needs of residents and fellows across the United States.^26^ The survey consisted of 40 questions in 5 subsections: Demographics, Residency Policies, Infertility Support, Fertility Preservation Support, and Fertility and Residency. Each question was multiple choice with some “Select all that apply” questions, and respondents could opt out of answering any question. Participants were informed their responses were anonymous. The total number of responses was 299. The complete survey is available in Supplementary Appendix SA1.

Percentages of each response out of the total respondents to the entire survey were calculated, as well as the total number of respondents to a particular question. Because of the sensitive nature of the topics covered in the survey, all of the questions were optional, which resulted in many responses missing at least an answer. We did not ultimately exclude any of the survey responses because we wanted to present all responses completely but have included how many responses we received for each question, as well as the number of responses for each question that were declined to answer. All percentages presented in the article were calculated from the total number of respondents to the entire survey.

Bivariate analysis using logistic regression was performed to test the relationship between predictor and outcome variables. Predictor variables were classified into categorical variables and chosen *a priori*. Multivariable logistic regression was then used to determine factors associated with support or nonsupport of infertility or fertility preservation using Stata version 5.1. The covariates included specialty, region, age, gender, marital status, parental status, and whether the person had a child during training.

The outcomes examined were perceived program and personal level of support for trainees dealing with infertility or undergoing fertility preservation—responses of “very” or “somewhat supportive” were grouped as supportive, while responses of “minimally” and “not supportive” were labeled unsupportive.

Association was also calculated between support level, respondent demographics—age, gender, marital status, whether they had children, and whether they had children during training—and program demographics, including surgical versus nonsurgical specialty, program region, and whether the program was in a state mandating fertility insurance coverage. To create binaries, age was categorized into older than and younger than 50 years, and marital status was separated into partnered (responses of married and partnered) and unpartnered (responses of divorced, single, or other).

## Results

### Demographics

Out of 299 respondents, the most represented specialties were emergency medicine (11.0%) and obstetrics and gynecology (9.7%). A total of 49.5% of respondents were female and were most commonly aged 40–49 (39.1%). A total of 70.4% of respondents were Caucasian. Most PDs were married (80.3%) and had children (80.6%). Of the respondents who reported having children, 51.0% had their children in training.

The majority of programs overseen by respondents had 20 or fewer trainees (52.5%), and most programs lasted three (40.8%) years. The residency programs were distributed across the United States, and 28.8% of the programs were located in states mandating fertility coverage (See Table 1 for complete respondent demographics and Supplementary Appendix Table SA2 for complete data tables).

**Table 1.** Demographic Characteristics of Surveyed Program Directors

Demographics	*N*	%	% (excluding not answered/not applicable)
Specialty			
Allergy and immunology	6	2.0%	2.0%
Anesthesiology	15	5.0%	5.0%
Dermatology	13	4.4%	4.4%
Emergency medicine	33	11.0%	11.0%
Endocrinology	19	6.4%	6.4%
Family medicine	27	9.0%	9.0%
Gastroenterology	10	3.3%	3.3%
General surgery	16	5.4%	5.4%
Internal medicine	20	6.7%	6.7%
Neurological surgery	2	0.7%	0.7%
Obstetrics and gynecology	29	9.7%	9.7%
Ophthalmology	14	4.7%	4.7%
Orthopedic surgery	7	2.3%	2.3%
Other	11	3.7%	3.7%
Other surgical subspecialty	1	0.3%	0.3%
Otolaryngology	7	2.3%	2.3%
Pathology	6	2.0%	2.0%
Pediatrics	12	4.0%	4.0%
Physical medicine and rehabilitation	4	1.3%	1.3%
Plastic surgery	3	1.0%	1.0%
Psychiatry	10	3.3%	3.3%
Radiation oncology	1	0.3%	0.3%
Radiology (diagnostic)	13	4.4%	4.4%
Thoracic surgery	5	1.7%	1.7%
Urology	15	5.0%	5.0%
Region			
Midwest	65	21.7%	22.1%
Northeast	96	32.1%	32.7%
South	84	28.1%	28.6%
West	44	14.7%	15.0%
Other	5	1.7%	1.7%
Not answered/not applicable	5	1.7%	
Total no. of residents/fellows			
<20	157	52.5%	52.9%
21–50	105	35.1%	35.5%
51–99	28	9.4%	9.4%
>100	7	2.3%	2.4%
Not answered/not applicable	2	0.7%	
Length of residency (years)			
2	39	13.0%	13.2%
3	122	40.8%	41.2%
4	82	27.4%	27.7%
5	42	14.1%	14.2%
6	7	2.3%	2.4%
>7	4	1.3%	1.4%
Not answered/not applicable	3	1.0%	
Age			
<30	6	2.0%	2.0%
30–39	52	17.4%	17.6%
40–49	117	39.1%	39.5%
50–59	68	22.7%	23.0%
60–69	46	15.4%	15.5%
>70	7	2.3%	2.4%
Not answered/not applicable	3	1.0%	
Gender			
Female	148	49.5%	50.2%
Male	145	48.5%	49.2%
Other	2	0.7%	0.7%
Not answered/not applicable	4	1.3%	
Race/ethnicity			
American Indian/Alaska Native	1	0.3%	0.4%
Asian/Pacific Islander	35	11.7%	12.1%
Black/African American	12	4.0%	4.2%
Caucasian	210	70.4%	72.7%
Latino/Hispanic	12	4.0%	4.2%
2 or more races	9	3.0%	3.1%
Other	10	3.3%	3.5%
Not answered/not applicable	10	3.3%	
Marital status			
Divorced	11	3.68%	3.77%
Married	240	80.27%	82.19%
Partnered	10	3.34%	3.42%
Single	26	8.70%	8.90%
Widowed	2	0.67%	0.68%
Other	3	1.00%	1.03%
Not answered/not applicable	7	2.34%	
Have children			
No	51	17.1%	17.5%
Yes	241	80.6%	82.5%
Not answered/not applicable	7	2.3%	
If yes, did you have your children while in residency or fellowship?			
No	123	51.0%	51.5%
Yes	116	48.1%	48.5%
Not answered/not applicable	2	0.8%	
Live in a state where fertility coverage is mandated^a^			
No	205	68.6%	70.5%
Yes	86	28.8%	29.5%
Not answered/not applicable	8	2.7%	

^a^ As of 2018, these states are Arkansas, Connecticut, Hawaii, Illinois, Louisiana, Maryland, Massachusetts, Montana, New Jersey, New York, Ohio, Rhode Island, and West Virginia.

### Residency policies

Parental leave policies varied between programs, but the most common lengths of leave allowed were 6–8 weeks of maternity (30.4%) and under 2 weeks of paternity leave (33.1%), with the possibility of extending time allowed in 62.5% of programs. During parental leave, the majority of missed work is covered by other residents (81.9%), as well as nonresidents, such as attendings and nurse practitioners (27.4%).

Most respondents lacked information on their program's insurance policies regarding infertility treatment and fertility preservation, with 57.2% not knowing coverage of infertility treatment and 68.6% not knowing policies for fertility preservation. Of those surveyed who did know their program's coverage, only 7.7% of programs provided full coverage for infertility treatment, and 1.7% fully covered fertility cryopreservation.

### Infertility support

Over half (52.2%) of PDs stated that none of their residents had ever disclosed facing infertility or RPL, although 55.2% estimated at least 5% of their residents were facing infertility or RPL. For residents with infertility, the most common resources offered were moral support from PDs (68.2%), time off for appointments (65.2%), and insurance coverage (36.1%).

**FIG. 1.**
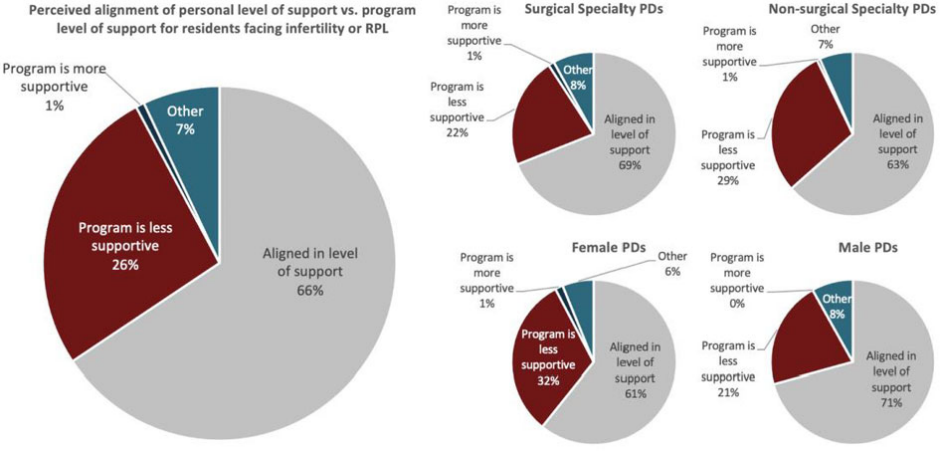
Perceived alignment of personal versus program level of support for residents with infertility or RPL unstratified and stratified by surgical versus nonsurgical specialty PDs and male versus female PDs. PDs, program directors; RPL, recurrent pregnancy loss.

Programs allowing their residents to take time off for treatment typically gave at least 2 days off each year (63.9%), which was primarily given on a case-by-case basis (75.6%). Overall, most PDs felt their personal level of support for residents facing infertility aligned with their program's level of support (56.2%). Those who felt the support levels were unaligned typically felt their program was less supportive (22.7%), as illustrated in Figure 1.

### Fertility preservation support

**FIG. 2.**
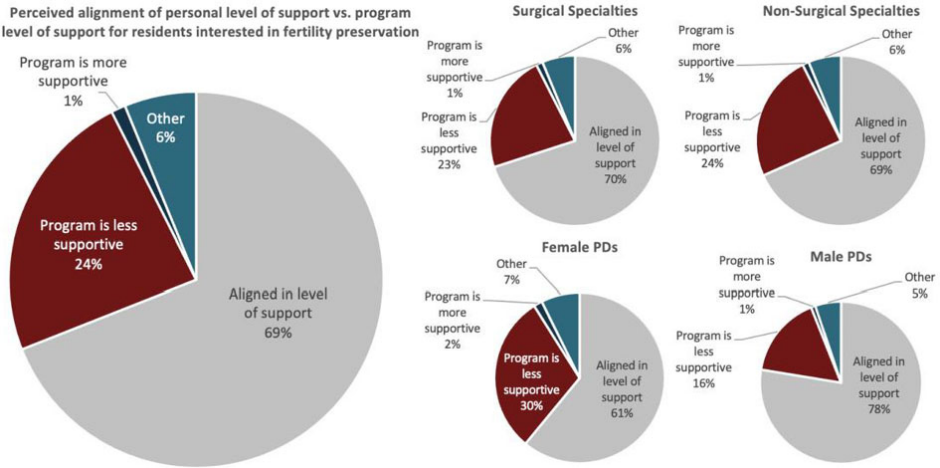
Perceived alignment of personal versus program level of support for residents interested in fertility preservation unstratified and stratified by surgical versus nonsurgical specialty PDs and male versus female PDs.

A total of 66.2% reported that none of their residents had ever expressed interest in fertility preservation to them. A total of 71.2% stated that, to their knowledge, none had undergone cryopreservation. For residents who did express interest, PDs stated that common resources included moral support from PDs (59.2%) and time off for appointments (48.5%). Residents were mostly granted time off on a case-by-case basis (72.2%), and 61.2% of programs gave residents at least 2 days off each year. A total of 55.9% of PDs believed their personal level of support for residents interested in fertility preservation was aligned with their program, and of respondents who felt support levels were unaligned, 19.1% felt their program was less supportive (Fig. 2).

### Fertility and residency

A total of 55.9% of residency PDs indicated that it was very or somewhat important to increase resources for residents undergoing fertility treatment, similar to 53.2% who felt the same for residents undergoing fertility preservation. Regarding residents' pregnancies, 25.4% of PDs strongly or somewhat encouraged it, and 43.1% stated they neither encouraged nor discouraged it; 74.6% said this stance did not differ between male and female trainees. A total of 55.9% of PDs agreed that a discount would alleviate the costs of infertility treatment, and 56.5% felt the same regarding cryopreservation.

Time (32.1%) and costs (24.8%) were seen as the two biggest hindrances to residents pursuing fertility treatments. PDs were also asked what should be changed to better support residents' reproductive needs, and the responses were as follows: “increasing awareness of individual needs” (47.5%), “official policies on fertility treatment” (34.1%), “financial support” (33.4%), “time off for treatment” (31.1%), “counseling” (24.4%), “reach out to department leadership, GME, or dean's office” (18.4%), and “nothing” (7.7%).

### Multivariate analysis

Bivariate analysis of each covariate was performed, and the predictor variables were not found to be statistically significantly correlated with support or nonsupport of infertility or fertility preservation services. Complete data tables from the multivariate analysis can be found in Supplementary Appendix Table SA3.

### Stratified results

#### Surgical versus nonsurgical specialty

After stratifying responses by PDs in surgical and nonsurgical specialties, the demographics of the two groups were similar. However, they reported differing maternity leave policies, with 67.0% of surgical specialties granting 4–8 weeks, while 62.1% of nonsurgical specialties granted 6–12 weeks. Nonsurgical PDs were more likely to state they were “very supportive” of their residents facing infertility, 82.2% compared with 74.7% of surgical PDs, and more surgery PDs did not give residents time off for infertility treatment (23.2% compared with 16.9%).

When surveyed about fertility preservation support, more surgery PDs (24.7%) reported that residents had expressed interest to them in fertility preservation than nonsurgery PDs (17.1%). Nonsurgical PDs (76.7%) stated that they felt they were “very supportive” of residents interested in fertility preservation, similar to surgical PDs (69.1%). Similar rates of surgery (22.7%) and nonsurgery programs (20.0%) did not allow residents to take time off for fertility preservation.

More surgery PDs felt it was “very important” to increase resources for residents undergoing infertility treatment and fertility preservation compared with nonsurgery PDs; however, more nonsurgical PDs “strongly supported” their residents getting pregnant. In addition, more surgery residency PDs “strongly agreed” that trainee discounts would help with the costs of treatment and cryopreservation. Both groups most commonly agreed that time and finances were the biggest barriers to residents pursuing fertility treatments and that the best ways to improve support were to increase their personal awareness of individual needs and to have official fertility treatment policies.

#### Male versus female

Stratifying data by male and female respondents showed roughly equal representation (148 women and 145 men). Of the respondents with children, 50.8% of men and 46% of women had children during their residency. While distributions of maternity leave lengths were similar, more male PDs reported giving <2 weeks of paternity leave, compared with female PDs (41.5% vs. 32.8%). In addition, male PDs were more likely to state that they did not know about residency insurance coverage of infertility treatment and fertility preservation.

Over two-thirds (67.7%) of male PDs reported that none of their residents had told them about facing infertility or RPL, compared with half (51.9%) of female PDs, and were more likely to estimate that none of their residents had infertility. While the majority of both groups stated that they felt that their personal level of support for trainees facing infertility was aligned with their program's support, 31.5% of female PDs felt their program was less supportive than they were, compared with 21.1% of males.

The majority of both male (83.1%) and female (77.6%) PDs stated that none of their trainees had expressed interest to them in fertility preservation, but most commonly offered time off and moral support for their residents. Most male respondents (57.4%) stated that their program was “very supportive” of residents interested in fertility preservation, while most female respondents (48.4%) reported their program was “somewhat supportive.”

More female PDs (32.5%) believed it was “very important” to increase resources for residents undergoing infertility treatment, compared with 16.2% of male PDs. Similarly, 30.3% of female PDs felt it was “very important” to increase resources for residents undergoing fertility preservation, whereas 16.4% of male PDs agreed. In addition, 38.8% of female respondents “strongly encouraged” pregnancy, but only 24.3% of male respondents did so. Most respondents from both groups stated their opinion did not differ between their male and female trainees, but female PDs (5.8%) were more likely to discourage their female residents from getting pregnant. More female PDs strongly agreed a trainee discount would help with the costs of assisted reproductive technologies.

Both groups agreed time and finances were the biggest barriers to trainees pursuing fertility treatments but differed in what they felt should be improved. Male respondents most commonly felt that increasing personal awareness regarding individual needs (59.1%) and financial support (37.3%) should be improved, while female respondents most commonly stated that personal awareness (62.8%) and time off for appointments (55.4%) needed improvement. A total of 13.6% of male PDs believed nothing currently needed improvement, compared with 6.6% of female PDs.

## Discussion

Due to the time-consuming nature of residency, age-related fertility decline is highly relevant for trainees who may then seek infertility treatment or fertility cryopreservation. Particularly because infertility has been previously determined to be prevalent at a higher rate in female physician than in the general population, this issue must be addressed during the postgraduate training period.^14^

While previous studies have examined this issue from the trainees' perspectives, confirming their interest in such treatments, this study is the first to determine PD opinions. Because PDs have influence over residents' access to these services, such as through determining financial support and establishing leave policies, it is important to examine their opinions to get a fuller perspective on how to improve the current situation.

Our study found variation in parental and medical leave policies for fertility treatments, highlighting the need for standardized practices. These differences could contribute to inequities in training, due to sex or specialty.^27^ In addition, our study found that only 55.9% of PDs felt it was important to increase resources for residents undergoing infertility treatment, and 53.2% said the same for residents undergoing fertility preservation. This inconsistent support may add stress onto an already rigorous training schedule, discouraging parenthood in fear of hindering one's career.^28,29^

Only 35.1% of PDs correctly identified age 35 as when female fertility decreases, and only 21.7% knew male fertility decreased around age 45, highlighting misinformation regarding reproduction, potentially impacting decision making on program policies. Most PDs also did not know their program's insurance coverage of fertility treatments. Trainees often turn to their PDs for guidance on policies, and lack of knowledge decreases the likelihood that trainees will consider those options for their reproductive needs.^25,30^

Most respondents stated none of their residents expressed interest in infertility treatment and estimated that the percentage of their residents facing infertility was around 5%–10%, consistent with a previous study's findings of a perceived lack of program support for residents with fertility concerns.^25^ However, given that infertility prevalence in the United States is 15.5% and previous studies stated at least 7%–8% infertility rate in residents,^25,31^ PDs may be underestimating the number of residents with infertility.

In addition, time off and moral support were the most common resources for residents undergoing infertility treatment, but few programs provided financial support, even though high costs are a major barrier for trainees.^32^ Trainees with infertility are burdened with medical school debt, low salary, and an intense schedule, further discouraging them from pursuing treatment.^33^

Similarly, the majority of respondents stated that none of their residents had expressed interest to them regarding fertility preservation and did not know any resident who had undergone the procedure. Most PDs were unaware of their program's insurance policy on fertility preservation but said that time off and moral support were the main resources for residents interested in cryopreservation. This lack of resources and knowledge regarding fertility cryopreservation may result in less dialogue between residents and PDs, discouraging them from pursuing treatment.

In a survey of OB/GYN residents, 29% of respondents considered fertility preservation, but only 2% sought consultation, revealing that residents actively choose not to try the procedure. Sixty-three percent of survey respondents had attributed their decision against fertility preservation to a lack of support, through scheduling and finances, from their program.^25^

Although only 1% of PDs discouraged residents from being pregnant, perceived lack of support can intimidate residents from approaching PDs with reproduction-related issues. Previous studies have suggested that PDs view pregnancies as detrimental to their programs: Sandler et al. found that general surgery PDs felt having children negatively impacted residents' work.^34^

A similar survey of surgery residents found that most residents did not feel supported by their programs during pregnancy, with 39% of respondents considering leaving the program and 30% advising future residents to not specialize in surgery if considering parenthood during training.^35^ However, PD support has been improving, according to a survey of residents across specialties from 2008 to 2015,^36^ as well as our data.

When asked about increasing resources for infertility treatment and fertility preservation, only a slight majority of residency PDs ranked the issue as important and agreed that a trainee discount would help with costs, compared with almost half of respondents who felt that increased support was not important. Time and finances were viewed by PDs as the primary barriers to pursuing treatments, the same reasons reported by trainees in prior surveys.^25,31^

The American Medical Women's Association Infertility Task Force has similarly called for reforms to address these hurdles to reproductive equity, advocating for more awareness of trainees' fertility issues, increased insurance coverage of reproductive treatments, and support for residents undergoing any fertility treatment.^37^ These positions are consistent with areas that PDs view as shortcomings in their support for trainees' reproductive needs, highlighting an opportunity for policy implementation and action.

Stratifying results by specialty revealed that nonsurgical PDs provided more time off for maternity leave and fertility treatments. Surgical PDs more commonly stated their residents expressed interest in fertility preservation to them, reflecting findings concluding that physicians are increasingly interested in oocyte cryopreservation due to their demanding careers.^38^ In addition, more surgery PDs felt it was necessary to improve resources to meet their residents' needs, such as through cost assistance.

Stratifying data by gender found that female PDs were more knowledgeable on their program's fertility insurance policies, as well as their residents' fertility issues, and were more likely to feel their program was not as supportive as they personally were with residents facing infertility. In addition to previous findings that female physicians retrospectively would have opted for more fertility support during their training,^14,20^ our findings also showed that female PDs emphasized the importance of increasing support for infertility treatment and fertility preservation more than males.

### Strengths and limitations

Strengths of the study include the in-depth survey questions, allowing for data on a broad range of subjects regarding fertility and residency. No other study has surveyed residency and fellowship PDs on their opinions regarding the fertility needs of their residents, and this study gave a new perspective on this issue from those who have the ability to affect change for residents. In addition, the survey reached a diverse variety of PDs, allowing us to stratify responses by gender and specialty.

As discussed in the Methods section, limitations of the study include that it was not possible to determine the response rate of the study because of indirect survey distribution methods, and that the number of respondents is small compared with the number of individuals the survey was sent to. The low response rate of this study means the results are a snapshot of PDs and their opinions, and the decision to conduct a population study of PDs, rather than selecting a sample, was based on the pilot study, which was a population study of residents and fellows.

Participation in the survey was optional, which may contribute to selection bias in respondents. This bias is potentially evident in the fact that more than half of respondents were female, a much larger percentage than the proportion of existing female PDs, which could have potentially skewed the results more favorably because these PDs were likely more supportive.^39–41^

Selection bias based on specialty also could have impacted the results. Of the programs we contacted, 5.0% were from emergency medicine specialties, and 6.0% were PDs in obstetrics and gynecology. Given that emergency medicine and obstetrics and gynecology comprised 11.0% and 9.7% of respondents, respectively, their relatively disproportionate representation within the survey's respondents could also have impacted the results.

Similarly, the specialties with the largest number of programs that we contacted, family medicine and internal medicine, were also not proportionately represented within the survey's respondents. In addition, the survey did not assess the length that a respondent had been a PD, and their responses to the survey questions could be affected by the amount of experience they have had in their position.

There are potentially institutional variations between programs, based on GME office or state law, which were not accounted for in this study. Despite these limitations, we believe this study highlights important and novel findings about PDs' perspectives and potential influence on medical trainees' fertility.

## Conclusions

Wide gaps of knowledge and awareness exist among postgraduate medical PDs on the impact of age on fertility and mechanisms of support for trainees accessing infertility treatment and fertility preservation. Coupled with previous research, the findings of this study highlight the increasing need for improved PD awareness of fertility-related issues, program insurance coverage of infertility treatment and fertility preservation services, and improvement in pregnancy and parental policy.

These changes would help meet medical trainees' reproductive needs and create a more equitable workplace for aspiring parents. We believe future research and education should focus on increasing dialogue between PDs and residents regarding fertility, improving family planning resources from PDs and their programs, and increasing resident awareness of these resources.

## Supplementary Material

Supplemental data

Supplemental data

Supplemental data

## Presentations

These data were presented, in part, as an oral abstract presentation at the American Society for Reproductive Medicine Scientific Congress in October 2020.

## Supplementary Material

Supplementary Appendix SA1

Supplementary Appendix Table SA2

Supplementary Appendix Table SA3

## Abbreviations Used

ACGME: Accreditation Council for Graduate Medical Education
PDs: program directors
RPL: recurrent pregnancy loss
