# Supplementary material for: Fertility Preservation and Infertility Treatment in Medical Training: An Assessment of Residency and Fellowship Program Directors' Attitudes
Source: Womens Health Rep (New Rochelle). 2021 Dec 7;2(1):576–85. doi: 10.1089/whr.2021.0044 (PMC8820404; doi:10.1089/whr.2021.0044)
Supplement: Supplemental data [file Suppl_AppendixSA1.docx]

**APPENDIX A – Survey**

I. Demographics

1. What specialty is your residency program in?
   1. Allergy and immunology
   2. Anesthesia
   3. Dermatology
   4. Emergency medicine
   5. Endocrinology
   6. Family medicine
   7. Internal medicine
   8. Gastroenterology
   9. General surgery
   10. Hematology
   11. Neurological surgery
   12. Neurology
   13. Obstetrics and gynecology
   14. Ophthalmology
   15. Orthopedic surgery
   16. Otolaryngology
   17. Pathology
   18. Pediatrics
   19. Plastic surgery
   20. Physical medicine & rehabilitation
   21. Psychiatry
   22. Radiation oncology
   23. Radiology (diagnostic)
   24. Thoracic surgery
   25. Urology
   26. Other surgical subspecialty
   27. Other (please specify)
   28. Fellowship (please specify division)
2. What region is your program located?
   1. Northeast
   2. Midwest
   3. South
   4. West
   5. Other (please specify)
3. Do you live in a state where fertility coverage by insurance is mandated? (Note: As of 2018, these states are Arkansas, Connecticut, Hawaii, Illinois, Louisiana, Maryland, Massachusetts, Montana, New Jersey, New York, Ohio, Rhode Island, and West Virginia)
   1. Yes
   2. No
4. How many total residents do you oversee?
   1. <20
   2. 21-50
   3. 51-99
   4. 100 and over
5. How old are you?
   1. < 30
   2. 30-39
   3. 40-49
   4. 50-59
   5. 60-69
   6. 70+
6. What is your gender?
   1. Male
   2. Female
   3. Other
7. With what race/ethnicity do you identify? (Check all that apply)
   1. Caucasian
   2. Black/African American
   3. American Indian/Alaska Native
   4. Asian/Pacific Islander
   5. Latino/Hispanic
   6. Other (please specify)
8. What is your current marital status?
   1. Married
   2. Partnered
   3. Single
   4. Divorced
   5. Other (please specify)
9. Do you have children?
   1. Yes
   2. No
10. If yes, did you have your children while in residency or fellowship?
    1. Yes
    2. No

II. Residency Policies

1. What is your residency program’s policy on the amount of maternity leave granted to female residents?
   1. < 2 weeks
   2. 2- <4 weeks
   3. 4 - <6 weeks
   4. 6 - < 8 weeks
   5. 8 to <12 weeks
   6. Greater than 12 weeks
2. What is your residency program’s policy on the amount of paternity leave granted to male residents?
   1. < 2 weeks
   2. 2- <4 weeks
   3. 4 - <6 weeks
   4. 6 - < 8 weeks
   5. 8 to <12 weeks
   6. Greater than 12 weeks
3. During parental leave, how is the **majority** of missed work covered? (Check all that apply as significant coverage)
   1. By other residents
   2. By extra non-resident help (i.e. attendings, advanced practice providers, etc.)
   3. Resident taking parental leave makes up missed calls
   4. Other (please specify)
4. Are residents allowed to extend maternity leave beyond a set amount (excluding emergency or medically indicated situations)?
   1. Yes
   2. No
   3. Other (please specify)
5. What is the residency insurance (offered through the GME) coverage of infertility treatment at your program?
   1. Covers all aspects of treatment
   2. Covers some aspects of treatment
   3. Not covered
   4. I don’t know
6. What is your residency program’s insurance coverage of fertility preservation (egg or embryo freezing)?
   1. Covers all aspects of treatment
   2. Covers some aspects of treatment
   3. Not covered
   4. I don’t know

III. Infertility Support

1. How many of your residents have disclosed to you that they are facing infertility or recurrent pregnancy loss?
   1. None
   2. <5%
   3. 6-10%
   4. 11-25%
   5. 25-50%
   6. 50%+
2. What is your estimate of how many residents in your program are facing infertility or recurrent pregnancy loss (RPL, defined as 2 or more miscarriages)?
   1. None
   2. <5%
   3. 6-10%
   4. 11-25%
   5. 26-50%
   6. 50%
3. In your opinion, has the number of residents with fertility issues changed in the past years?
   1. Increased
   2. Decreased
   3. No changed
   4. Don’t know
4. To your knowledge, how many residents in your program have undergone fertility treatment, such as IUI or IVF?
   1. None
   2. <5%
   3. 6-10%
   4. 11-25%
   5. 26-50%
   6. 50%
5. What resources exist in your residency program for residents facing infertility/recurrent pregnancy loss? (Check all that apply)
   1. Insurance coverage
   2. Trainee discount
   3. Other financial support
   4. Time off for appointments
   5. Moral support provided by program director
   6. Other (please specify)
6. **How supportive do you feel your program is** towards residents with fertility issues (infertility or RPL)?
   1. Very supportive
   2. Somewhat supportive
   3. Minimally supportive
   4. Not supportive
7. **How supportive do you feel personally** towards residents with fertility issues (infertility or RPL)?
   1. Very supportive
   2. Somewhat supportive
   3. Minimally supportive
   4. Not supportive
8. What is your attitude towards the alignment of your personal level of support and program level of support for residents facing infertility or RPL?
   1. My program and I are aligned in level of support
   2. My program is more supportive than I personally feel
   3. My program is less supportive than I personally feel
   4. Other (explain)
9. Are there measures currently in place to improve support for residents facing infertility or RPL?
   1. Yes (please explain)
   2. No
   3. Other (please explain)
10. Are residents allowed to take time off for treatment for infertility or RPL?
    1. No
    2. Yes - 2 days or less per year
    3. Yes - 3 days to 1 week per year
    4. Yes - greater than 1 week per year
11. Do you have any official or standardized policies on taking time off for infertility and RPL treatment?
    1. Yes
    2. No - case by case basis
    3. Other (please explain)

IV. Fertility Preservation

1. How many of your residents have expressed interest to you in fertility preservation?
   1. None
   2. <5%
   3. 6-10%
   4. 11-25%
   5. 26-50%
   6. 50%
2. To your knowledge, how many of your residents have undergone fertility preservation?
   1. None
   2. <5%
   3. 6-10%
   4. 11-25%
   5. 25-50%
   6. 50%
3. What resources exist in your residency program for residents interested in fertility preservation? (Check all that apply)
   1. Insurance coverage
   2. Trainee discount
   3. Other financial support
   4. Time off for appointments
   5. Moral support provided by program director
   6. Other (please specify)
4. **How supportive do you feel your program is** towards residents interested in fertility preservation?
   1. Very supportive
   2. Somewhat supportive
   3. Minimally supportive
   4. Not supportive
5. **How supportive do you feel personally** towards residents interested in fertility preservation?
   1. Very supportive
   2. Somewhat supportive
   3. Minimally supportive
   4. Not supportive
6. What is your attitude towards the alignment of your personal level of support and program level of support for residents interested in fertility preservation?
   1. My program and I are aligned in level of support
   2. My program is more supportive than I personally feel
   3. My program is less supportive than I personally feel
   4. Other (explain)
7. Are there measures currently in place to improve support for residents interested in fertility preservation?
   1. Yes (please explain)
   2. No
   3. Other (please explain)
8. Are residents allowed to take time off for treatment for fertility preservation?
   1. No
   2. Yes - 2 days or less per year
   3. Yes - 3 days to 1 week per year
   4. Yes - Greater than 1 week per year
9. Do you have any official or standardized policies on taking time off for fertility preservation?
   1. Yes
   2. No - case by case basis
   3. Other (please explain)

V. Fertility & Residency

1. What is your understanding of the effect of age on fertility in women?
   1. No effect
   2. Fertility decreases around age 25
   3. Fertility decreases around age 30
   4. Fertility decreases around age 35
   5. Fertility decreases around age 40
   6. Other (please specify)
2. What is your understanding of the effect of age on fertility in men?
   1. No effect
   2. Fertility decreases around age 30
   3. Fertility decreases around age 35
   4. Fertility decreases around age 40
   5. Fertility decreases around age 45
   6. Other (please specify)
3. How important do you feel it is to increase resources for residents undergoing infertility or RPL treatment?
   1. Very important
   2. Somewhat important
   3. Neither important nor unimportant
   4. Somewhat unimportant
   5. Very unimportant
4. How important do you feel it is to increase resources for residents undergoing fertility preservation?
   1. Very important
   2. Somewhat important
   3. Neither important nor unimportant
   4. Somewhat unimportant
   5. Very unimportant
5. As a residency director, what is your stance regarding residents trying to get pregnant?
   1. Strongly encourage
   2. Somewhat encourage
   3. Neither encourage nor discourage
   4. Somewhat discourage
   5. Strongly discourage
6. Does your opinion on the above differ for male and female residents?
   1. No
   2. Yes - “discourage” female residents more
   3. Yes - “discourage” male residents more
   4. Yes - other
   5. Other (please specify)
7. A trainee discount would help with the costs of undergoing assisted reproductive technologies in order to conceive.
   1. Strongly agree
   2. Somewhat agree
   3. Neither agree nor disagree
   4. Somewhat disagree
   5. Strongly disagree
   6. No opinion
8. A trainee discount would help with the costs of undergoing fertility preservation (egg or embryo freezing).
   1. Strongly agree
   2. Somewhat agree
   3. Neither agree nor disagree
   4. Somewhat disagree
   5. Strongly disagree
   6. No opinion
9. In your opinion, what is the biggest barrier to pursuing fertility treatments while in training?
   1. Time
   2. Finances
   3. Lack of information
   4. Lack of partner
   5. Emotional reasons
   6. Geographical reasons (i.e. partner in different location)
   7. Other, please specify
   8. No opinion
10. What do you think should be improved in the current situation in the program regarding support to residents who may be struggling with infertility or interested in fertility preservation? (Check all that apply)
    1. Increasing personal awareness of individual needs
    2. Financial support
    3. Official policies on fertility treatment
    4. Counseling
    5. Time off for fertility treatment
    6. Expressing personal support during orientation
    7. Reach out to department leadership, GME, or dean’s office
    8. Nothing
    9. Other (please specify)

This is the end of the survey. Please feel free to add any additional comments on infertility, fertility preservation in training or any other topics addressed in this survey. Thank you so much for your participation!
