# Supplementary material for: Fertility Preservation and Infertility Treatment in Medical Training: An Assessment of Residency and Fellowship Program Directors' Attitudes
Source: Womens Health Rep (New Rochelle). 2021 Dec 7;2(1):576–85. doi: 10.1089/whr.2021.0044 (PMC8820404; doi:10.1089/whr.2021.0044)
Supplement: Supplemental data [file Suppl_Appendix_TableSA2.docx]

**APPENDIX B – Data Tables**

**Unstratified Data**

Total N = 299

| **Table I. Demographics** | | **N** | **Percentage** | **Percentage (excluding Not Answered/Not Applicable)** |
| --- | --- | --- | --- | --- |
| Specialty |  |  |  |  |
|  | Allergy and immunology | 6 | 2.0% | 2.0% |
|  | Anesthesiology | 15 | 5.0% | 5.0% |
|  | Dermatology | 13 | 4.4% | 4.4% |
|  | Emergency medicine | 33 | 11.0% | 11.0% |
|  | Endocrinology | 19 | 6.4% | 6.4% |
|  | Family medicine | 27 | 9.0% | 9.0% |
|  | Gastroenterology | 10 | 3.3% | 3.3% |
|  | General surgery | 16 | 5.4% | 5.4% |
|  | Internal medicine | 20 | 6.7% | 6.7% |
|  | Neurological surgery | 2 | 0.7% | 0.7% |
|  | Obstetrics and gynecology | 29 | 9.7% | 9.7% |
|  | Ophthalmology | 14 | 4.7% | 4.7% |
|  | Orthopedic surgery | 7 | 2.3% | 2.3% |
|  | Other | 11 | 3.7% | 3.7% |
|  | Other surgical subspecialty | 1 | 0.3% | 0.3% |
|  | Otolaryngology | 7 | 2.3% | 2.3% |
|  | Pathology | 6 | 2.0% | 2.0% |
|  | Pediatrics | 12 | 4.0% | 4.0% |
|  | Physical medicine & rehabilitation | 4 | 1.3% | 1.3% |
|  | Plastic surgery | 3 | 1.0% | 1.0% |
|  | Psychiatry | 10 | 3.3% | 3.3% |
|  | Radiation oncology | 1 | 0.3% | 0.3% |
|  | Radiology (diagnostic) | 13 | 4.4% | 4.4% |
|  | Thoracic surgery | 5 | 1.7% | 1.7% |
|  | Urology | 15 | 5.0% | 5.0% |
| Region |  |  |  |  |
|  | Midwest | 65 | 21.7% | 22.1% |
|  | Northeast | 96 | 32.1% | 32.7% |
|  | South | 84 | 28.1% | 28.6% |
|  | West | 44 | 14.7% | 15.0% |
|  | Other | 5 | 1.7% | 1.7% |
|  | Not Answered/Not Applicable | 5 | 1.7% |  |
| Total Number of Residents/Fellows | |  |  |  |
|  | <20 | 157 | 52.5% | 52.9% |
|  | 21-50 | 105 | 35.1% | 35.5% |
|  | 51-99 | 28 | 9.4% | 9.4% |
|  | >100 | 7 | 2.3% | 2.4% |
|  | Not Answered/Not Applicable | 2 | 0.7% |  |
| Length of residency (years) | |  |  |  |
|  | 2 | 39 | 13.0% | 13.2% |
|  | 3 | 122 | 40.8% | 41.2% |
|  | 4 | 82 | 27.4% | 27.7% |
|  | 5 | 42 | 14.1% | 14.2% |
|  | 6 | 7 | 2.3% | 2.4% |
|  | >7 | 4 | 1.3% | 1.4% |
|  | Not Answered/Not Applicable | 3 | 1.0% |  |
| Age |  |  |  |  |
|  | <30 | 6 | 2.0% | 2.0% |
|  | 30-39 | 52 | 17.4% | 17.6% |
|  | 40-49 | 117 | 39.1% | 39.5% |
|  | 50-59 | 68 | 22.7% | 23.0% |
|  | 60-69 | 46 | 15.4% | 15.5% |
|  | >70 | 7 | 2.3% | 2.4% |
|  | Not Answered/Not Applicable | 3 | 1.0% |  |
| Gender |  |  |  |  |
|  | Female | 148 | 49.5% | 50.2% |
|  | Male | 145 | 48.5% | 49.2% |
|  | Other | 2 | 0.7% | 0.7% |
|  | Not Answered/Not Applicable | 4 | 1.3% |  |
| Race/Ethnicity |  |  |  |  |
|  | American Indian/Alaska Native | 1 | 0.3% | 0.4% |
|  | Asian/Pacific Islander | 35 | 11.7% | 12.1% |
|  | Black/African American | 12 | 4.0% | 4.2% |
|  | Caucasian | 210 | 70.4% | 72.7% |
|  | Latino/Hispanic | 12 | 4.0% | 4.2% |
|  | 2 or more races | 9 | 3.0% | 3.1% |
|  | Other | 10 | 3.3% | 3.5% |
|  | Not Answered/Not Applicable | 10 | 3.3% |  |
| Marital Status |  |  |  |  |
|  | Divorced | 11 | 3.68% | 3.77% |
|  | Married | 240 | 80.27% | 82.19% |
|  | Partnered | 10 | 3.34% | 3.42% |
|  | Single | 26 | 8.70% | 8.90% |
|  | Widowed | 2 | 0.67% | 0.68% |
|  | Other | 3 | 1.00% | 1.03% |
|  | Not Answered/Not Applicable | 7 | 2.34% |  |
| Have children |  |  |  |  |
|  | No | 51 | 17.1% | 17.5% |
|  | Yes | 241 | 80.6% | 82.5% |
|  | Not Answered/Not Applicable | 7 | 2.3% |  |
| If yes, did you have your children while in residency or fellowship? | | |  |  |
|  | No | 123 | 51.0% | 51.5% |
|  | Yes | 116 | 48.1% | 48.5% |
|  | Not Answered/Not Applicable | 2 | 0.8% |  |
| Live in a state where fertility coverage is mandated* | | |  |  |
|  | No | 205 | 68.6% | 70.5% |
|  | Yes | 86 | 28.8% | 29.5% |
|  | Not Answered/Not Applicable | 8 | 2.7% |  |

*As of 2018, these states are Arkansas, Connecticut, Hawaii, Illinois, Louisiana, Maryland, Massachusetts, Montana, New Jersey, New York, Ohio, Rhode Island, and West Virginia

| **Table II. Residency Policies** | | **N** | **Percentage** | **Percentage (excluding Not Answered/Not Applicable)** |
| --- | --- | --- | --- | --- |
| Amount of maternity leave granted to female residents | |  |  |  |
|  | <2 weeks | 5 | 1.7% | 1.9% |
|  | 2- <4 weeks | 11 | 3.7% | 4.1% |
|  | 4 - <6 weeks | 61 | 20.4% | 22.9% |
|  | 6 - <8 weeks | 91 | 30.4% | 34.1% |
|  | 8 to <12 weeks | 68 | 22.7% | 25.5% |
|  | 12+ weeks | 31 | 10.4% | 11.6% |
|  | Not Answered/Not Applicable | 32 | 10.7% |  |
| Amount of paternity leave granted to male residents | |  |  |  |
|  | <2 weeks | 99 | 33.1% | 37.6% |
|  | 2- <4 weeks | 53 | 17.7% | 20.2% |
|  | 4 - <6 weeks | 28 | 9.4% | 10.7% |
|  | 6 - <8 weeks | 31 | 10.4% | 11.8% |
|  | 8 to <12 weeks | 37 | 12.4% | 14.1% |
|  | 12+ weeks | 15 | 5.0% | 5.7% |
|  | Not Answered/Not Applicable | 36 | 12.0% |  |
| During parental leave, how is the majority of missed work covered? (Select all that apply) | | | | |
|  | By other residents | 245 | 81.9% | 89.7% |
|  | By extra non-resident help | 82 | 27.4% | 30.0% |
|  | Resident taking parental leave makes up missed calls | 59 | 19.7% | 21.6% |
|  | Other | 16 | 5.4% | 5.9% |
|  | Not Answered/Not Applicable | 26 | 8.7% |  |
| 2 | | | | |
|  | Yes | 187 | 62.5% | 68.3% |
|  | No | 49 | 16.4% | 17.9% |
|  | Other | 38 | 12.7% | 13.9% |
|  | Not Answered/Not Applicable | 25 | 8.4% |  |
| What is the residency insurance (offered through the GME) coverage of infertility treatment at your program? | | | | |
|  | Covers all aspects of treatment | 23 | 7.7% | 8.4% |
|  | Covers some aspects of treatment | 43 | 14.4% | 15.6% |
|  | I don’t know | 171 | 57.2% | 62.2% |
|  | Not covered | 38 | 12.7% | 13.8% |
|  | Not Answered/Not Applicable | 24 | 8.0% |  |
| What is your residency program’s insurance coverage of fertility preservation (egg or embryo freezing)? | | | | |
|  | Covers all aspects of treatment | 5 | 1.7% | 1.8% |
|  | Covers some aspects of treatment | 8 | 2.7% | 2.9% |
|  | Don’t know | 205 | 68.6% | 74.0% |
|  | Not covered | 59 | 19.7% | 21.3% |
|  | Not Answered/Not Applicable | 22 | 7.4% |  |

| **Table III. Infertility Support** | | **N** | **Percentage** | **Percentage (excluding Not Answered/Not Applicable)** |
| --- | --- | --- | --- | --- |
| How many of your residents have disclosed to you that they are facing infertility or recurrent pregnancy loss (RPL, 2 or more miscarriages)? | | | | |
|  | None | 156 | 52.2% | 60.0% |
|  | <5% | 70 | 23.4% | 26.9% |
|  | 6-10% | 26 | 8.7% | 10.0% |
|  | 11-25% | 6 | 2.0% | 2.3% |
|  | >26% | 2 | 0.8% | 0.8% |
|  | Not Answered/Not Applicable | 39 | 13.0% |  |
| What is your estimate of how many residents in your program are facing infertility or recurrent pregnancy loss (RPL, defined as 2 or more miscarriages)? | | | | |
|  | None | 90 | 30.1% | 35.3% |
|  | <5% | 92 | 30.8% | 36.1% |
|  | 6-10% | 60 | 20.1% | 23.5% |
|  | 11-25% | 11 | 3.7% | 4.3% |
|  | >26% | 2 | 0.8% | 0.8% |
|  | Not Answered/Not Applicable | 44 | 14.7% |  |
| In your opinion, has the number of residents with fertility issues changed in the past years? | | | | |
|  | Decreased | 1 | 0.3% | 1.0% |
|  | Increased | 35 | 11.7% | 33.3% |
|  | No change | 69 | 23.1% | 65.7% |
|  | Not Answered/Not Applicable | 194 | 64.9% |  |
| To your knowledge, how many residents in your program have undergone fertility treatment, such as IUI or IVF? | | | | |
|  | None | 158 | 52.8% | 62.0% |
|  | <5% | 65 | 21.7% | 25.5% |
|  | 6-10% | 25 | 8.4% | 9.8% |
|  | 11-25% | 6 | 2.0% | 2.4% |
|  | >26% | 1 | 0.3% | 0.4% |
|  | Not Answered/Not Applicable | 44 | 14.7% |  |
| What resources exist in your residency program for residents facing infertility/recurrent pregnancy loss? (Check all that apply) | | | | |
|  | Time off for appointments | 195 | 65.2% | 80.6% |
|  | Moral support from program director | 204 | 68.2% | 84.3% |
|  | Trainee discount | 12 | 4.0% | 5.0% |
|  | Insurance coverage | 108 | 36.1% | 44.6% |
|  | Other financial support | 3 | 1.0% | 1.2% |
|  | Other | 25 | 8.36% | 10.3 |
|  | Not Answered/Not Applicable | 57 | 19.1% |  |
| How supportive do you feel your program is towards residents with fertility issues? | | | | |
|  | Very supportive | 125 | 41.8% | 49.2% |
|  | Somewhat supportive | 101 | 33.8% | 39.8% |
|  | Minimally supportive | 21 | 7.0% | 8.3% |
|  | Not supportive | 7 | 2.3% | 2.8% |
|  | Not Answered/Not Applicable | 45 | 15.1% |  |
| How supportive do you feel personally towards residents with fertility issues? | | | | |
|  | Very supportive | 206 | 68.9% | 79.8% |
|  | Somewhat supportive | 46 | 15.4% | 17.8% |
|  | Minimally supportive | 5 | 1.7% | 1.9% |
|  | Not supportive | 1 | 0.3% | 0.4% |
|  | Not Answered/Not Applicable | 41 | 13.7% |  |
| What is your attitude towards the alignment of your personal level of support and program level of support for residents facing infertility or RPL? | | | | |
|  | Aligned in level of support | 168 | 56.2% | 65.6% |
|  | Program is less supportive | 68 | 22.7% | 26.6% |
|  | Program is more supportive | 2 | 0.7% | 0.8% |
|  | Other | 18 | 6.0% | 7.0% |
|  | Not Answered/Not Applicable | 43 | 14.4% |  |
| Are there measures currently in place to improve support for residents facing infertility or RPL? | | | | |
|  | Yes | 18 | 6.0% | 7.3% |
|  | No | 207 | 69.2% | 83.5% |
|  | Other | 23 | 7.7% | 9.3% |
|  | Not Answered/Not Applicable | 51 | 17.1% |  |
| Are residents allowed to take time off for treatment for infertility or RPL? | | | | |
|  | No | 46 | 15.4% | 19.4% |
|  | Yes - 2 days or less per year | 21 | 7.0% | 8.9% |
|  | Yes - 3 days to 1 week per year | 58 | 19.4% | 24.5% |
|  | Yes - greater than 1 week per year | 112 | 37.5% | 47.3% |
|  | Not Answered/Not Applicable | 62 | 20.7% |  |
| Do you have any official or standardized policies on taking time off for infertility and RPL treatment? | | | | |
|  | Yes | 6 | 2.0% | 2.4% |
|  | No - case by case basis | 226 | 75.6% | 89.7% |
|  | Other | 20 | 6.7% | 7.9% |
|  | Not Answered/Not Applicable | 47 | 15.7% |  |
| How many residents have taken time off for infertility or RPL treatment in your program? | | | | |
|  | None | 175 | 58.5% | 69.4% |
|  | <5% | 56 | 18.7% | 22.2% |
|  | 6-10% | 17 | 5.7% | 6.8% |
|  | >11% | 4 | 1.3% | 1.6% |
|  | Not Answered/Not Applicable | 47 | 15.7% |  |

| **Table IV. Fertility Preservation Support** | | **N** | **Percentage** | **Percentage (excluding Not Answered/Not Applicable)** |
| --- | --- | --- | --- | --- |
| How many of your residents have expressed interest to you in fertility preservation? | | | | |
|  | None | 198 | 66.2% | 80.5% |
|  | <5% | 33 | 11.0% | 13.4% |
|  | 6-10% | 13 | 4.4% | 5.3% |
|  | >11% | 2 | 0.7% | 0.8% |
|  | Not Answered/Not Applicable | 53 | 17.7% |  |
| To your knowledge, how many of your residents have undergone fertility preservation? | | | | |
|  | None | 213 | 71.2% | 86.9% |
|  | <5% | 30 | 10.0% | 12.2% |
|  | >6% | 2 | 0.7% | 0.8% |
|  | Not Answered/Not Applicable | 54 | 18.0% |  |
| What resources exist in your residency program for residents interested in fertility preservation? (Check all that apply) | | | | |
|  | Time off for appointments | 145 | 48.5% | 65.6% |
|  | Moral support from program director | 177 | 59.2% | 80.1% |
|  | Trainee discount | 6 | 2.0% | 2.7% |
|  | Insurance coverage | 56 | 18.7% | 25.3% |
|  | Other financial support | 3 | 1.00% | 1.4% |
|  | Other | 28 | 9.4% | 12.7% |
|  | Not Answered/Not Applicable | 78 | 26.1% |  |
| How supportive do you feel your program is towards residents interested in fertility preservation? | | | | |
|  | Very supportive | 115 | 38.5% | 47.9% |
|  | Somewhat supportive | 91 | 30.4% | 37.9% |
|  | Minimally supportive | 25 | 8.4% | 10.4% |
|  | Not supportive | 9 | 3.0% | 3.8% |
|  | Not Answered/Not Applicable | 54 | 18.1% |  |
| How supportive do you feel personally towards residents interested in fertility preservation? | | | | |
|  | Very supportive | 182 | 61.9% | 74.3% |
|  | Somewhat supportive | 53 | 17.7% | 21.6% |
|  | Minimally supportive | 9 | 3.0% | 3.7% |
|  | Not supportive | 1 | 0.3% | 0.4% |
|  | Not Answered/Not Applicable | 54 | 18.1% |  |
| What is your attitude towards the alignment of your personal level of support and program level of support for residents interested in fertility preservation? | | | | |
|  | Aligned in level of support | 167 | 55.9% | 69.0% |
|  | Program is less supportive | 57 | 19.1% | 23.6% |
|  | Program is more supportive | 3 | 1.0% | 1.2% |
|  | Other | 15 | 5.0% | 6.2% |
|  | Not Answered/Not Applicable | 57 | 19.1% |  |
| Are there measures currently in place to improve support for residents interested in fertility preservation? | | | | |
|  | Yes | 14 | 4.7% | 5.9% |
|  | No | 206 | 68.9% | 86.2% |
|  | Other | 19 | 6.4% | 8.0% |
|  | Not Answered/Not Applicable | 60 | 20.1% |  |
| Are residents allowed to take time off for treatment for fertility preservation? | | | | |
|  | No | 48 | 16.1% | 20.8% |
|  | Yes - 2 days or less per year | 33 | 11.0% | 14.3% |
|  | Yes - 3 days to 1 week per year | 53 | 17.7% | 22.9% |
|  | Yes - greater than 1 week per year | 97 | 32.4% | 42.0% |
|  | Not Answered/Not Applicable | 68 | 22.7% |  |
| Do you have any official or standardized policies on taking time off for fertility preservation? | | | | |
|  | Yes | 6 | 2.0% | 2.5% |
|  | No - case by case basis | 216 | 72.2% | 89.6% |
|  | Other | 19 | 6.4% | 7.9% |
|  | Not Answered/Not Applicable | 58 | 19.4% |  |
| How many residents have taken time off for fertility preservation treatment in your program? | | | | |
|  | None | 214 | 71.6% | 88.4% |
|  | <5% | 26 | 8.7% | 10.7% |
|  | 6-10% | 2 | 0.7% | 0.8% |
|  | Not Answered/Not Applicable | 57 | 19.1% |  |

| **Table V. Fertility & Residency** | | **N** | **Percentage** | **Percentage (excluding Not Answered/Not Applicable)** |
| --- | --- | --- | --- | --- |
| What is your understanding of the effect of age on fertility in women? | | | | |
|  | No effect | 0 | 0.0% | 0.0% |
|  | Fertility decreases around age 25 | 17 | 5.7% | 7.1% |
|  | Fertility decreases around age 30 | 97 | 3.24% | 40.6% |
|  | Fertility decreases around age 35 | 105 | 35.1% | 43.9% |
|  | Fertility decreases around age 40 | 12 | 4.0% | 5.0% |
|  | Other | 8 | 2.7% | 3.4% |
|  | Not Answered/Not Applicable | 60 | 20.1% |  |
| What is your understanding of the effect of age on fertility in men? | | | | |
|  | No effect | 65 | 21.7% | 27.4% |
|  | Fertility decreases around age 25 | 0 | 0.0% | 0.00% |
|  | Fertility decreases around age 35 | 14 | 4.7% | 5.9% |
|  | Fertility decreases around age 30 | 6 | 2.0% | 2.5% |
|  | Fertility decreases around age 40 | 73 | 24.4% | 30.8% |
|  | Fertility decreases around age 45 | 65 | 21.7% | 27.4% |
|  | Other | 14 | 4.7% | 5.9% |
|  | Not Answered/Not Applicable | 62 | 20.7% |  |
| How important do you feel it is to increase resources for residents undergoing infertility or RPL treatment? | | | | |
|  | Very important | 60 | 20.1% | 25.3% |
|  | Somewhat important | 107 | 35.8% | 45.2% |
|  | Neither important nor unimportant | 57 | 19.1% | 24.1% |
|  | Somewhat unimportant | 10 | 3.3% | 4.2% |
|  | Very unimportant | 3 | 1.0% | 1.3% |
|  | Not Answered/Not Applicable | 62 | 20.7% |  |
| How important do you feel it is to increase resources for residents undergoing fertility preservation? | | | | |
|  | Very important | 57 | 19.1% | 24.3% |
|  | Somewhat important | 102 | 34.1% | 43.4% |
|  | Neither important nor unimportant | 59 | 19.7% | 25.1% |
|  | Somewhat unimportant | 14 | 4.7% | 6.0% |
|  | Very unimportant | 3 | 1.0% | 1.3% |
|  | Not Answered/Not Applicable | 64 | 21.4% |  |
| As a residency director, what is your stance regarding residents trying to get pregnant? | | | | |
|  | Strongly encourage | 76 | 25.4% | 32.3% |
|  | Somewhat encourage | 27 | 9.0% | 11.5% |
|  | Neither encourage nor discourage | 129 | 43.1% | 54.9% |
|  | Somewhat discourage | 3 | 1.0% | 1.3% |
|  | Strongly discourage | 0 | 0.0% | 0.0% |
|  | Not Answered/Not Applicable | 64 | 21.4% |  |
| Does your opinion on the above differ for male and female residents? | | | | |
|  | No | 223 | 74.6% | 94.5% |
|  | Yes - discourage female residents more | 8 | 2.7% | 3.4% |
|  | Yes - discourage male residents more | 0 | 0.0% | 0.0% |
|  | Yes - other | 3 | 1.0% | 1.3% |
|  | Other | 2 | 0.7% | 0.9% |
|  | Not Answered/Not Applicable | 63 | 21.1% |  |
| A trainee discount would help with the costs of undergoing assisted reproductive technologies in order to conceive. | | | | |
|  | Strongly agree | 111 | 37.1% | 47.2% |
|  | Somewhat agree | 56 | 18.7% | 23.8% |
|  | Neither agree nor disagree | 29 | 9.7% | 12.3% |
|  | Somewhat disagree | 4 | 1.3% | 1.7% |
|  | Strongly disagree | 3 | 1.0% | 1.3% |
|  | No opinion | 32 | 10.7% | 13.6% |
|  | Not Answered/Not Applicable | 64 | 21.4% |  |
| A trainee discount would help with the costs of undergoing fertility preservation. | | | | |
|  | Strongly agree | 114 | 38.1% | 48.7% |
|  | Somewhat agree | 55 | 18.4% | 23.5% |
|  | Neither agree nor disagree | 26 | 8.7% | 11.1% |
|  | Somewhat disagree | 2 | 0.7% | 0.9% |
|  | Strongly disagree | 4 | 1.3% | 1.7% |
|  | No opinion | 33 | 11.0% | 14.1% |
|  | Not Answered/Not Applicable | 65 | 21.7% |  |
| In your opinion, what is the biggest barrier to pursuing fertility treatments while in training? | | | | |
|  | Time | 96 | 32.1% | 40.5% |
|  | Finances | 74 | 24.8% | 31.2% |
|  | Lack of information | 10 | 3.3% | 4.2% |
|  | Lack of partner | 9 | 3.0% | 3.8% |
|  | Emotional reasons | 3 | 1.0% | 1.4% |
|  | Other | 11 | 3.7% | 4.6% |
|  | No opinion | 34 | 11.4% | 14.4% |
|  | Not Answered/Not Applicable | 62 | 20.7% |  |
| What do you think should be improved in the current situation in the program regarding support to residents who may be struggling with infertility or interested in fertility preservation? (Check all that apply) | | | | |
|  | Counseling | 73 | 24.4% | 31.2% |
|  | Time off for fertility treatment | 93 | 31.1% | 39.7% |
|  | Expressing personal support during orientation | 64 | 12.8% | 27.4% |
|  | Financial support | 100 | 33.4% | 42.7% |
|  | Reach out to department leadership, GME, or dean’s office | 55 | 18.4% | 23.5% |
|  | Increasing personal awareness of individual needs | 142 | 47.5% | 60.7% |
|  | Official policies on fertility treatment | 102 | 34.1% | 43.6% |
|  | Nothing | 23 | 7.7% | 9.8% |
|  | Other | 8 | 2.7% | 3.4% |
|  | Not Answered/Not Applicable | 65 | 21.7% |  |

**Stratified – Female**

N = 148

| **Table 1. Demographics** | | **N** | **Percentage** | **Percentage (excluding Not Answered/Not Applicable)** |
| --- | --- | --- | --- | --- |
| Specialty | | | | |
|  | Allergy and immunology | 4 | 2.7% | 2.7% |
|  | Anesthesiology | 6 | 4.1% | 4.1% |
|  | Dermatology | 7 | 4.7% | 4.8% |
|  | Emergency medicine | 17 | 11.5% | 11.6% |
|  | Endocrinology | 9 | 6.1% | 6.1% |
|  | Family medicine | 12 | 8.1% | 8.2% |
|  | Gastroenterology | 3 | 2.0% | 2.0% |
|  | General surgery | 8 | 5.4% | 5.4% |
|  | Internal medicine | 9 | 6.1% | 6.1% |
|  | Neurological surgery | 1 | 0.7% | 0.7% |
|  | Obstetrics and gynecology | 24 | 16.2% | 16.3% |
|  | Ophthalmology | 6 | 4.1% | 4.1% |
|  | Orthopedic surgery | 1 | 0.7% | 0.7% |
|  | Other | 5 | 3.4% | 3.4% |
|  | Otolaryngology | 4 | 2.7% | 2.7% |
|  | Pathology | 4 | 2.7% | 2.7% |
|  | Pediatrics | 10 | 6.8% | 6.8% |
|  | Physical medicine & rehabilitation | 3 | 2.0% | 2.0% |
|  | Psychiatry | 3 | 2.0% | 2.0% |
|  | Radiation oncology | 1 | 0.7% | 0.7% |
|  | Radiology (diagnostic) | 5 | 3.4% | 3.4% |
|  | Urology | 5 | 3.4% | 3.4% |
|  | Not Answered/Not Applicable | 1 | 0.7% |  |
| Region |  | | | |
|  | Midwest | 30 | 20.3% | 20.7% |
|  | Northeast | 53 | 35.8% | 36.6% |
|  | South | 37 | 25.0% | 25.5% |
|  | West | 24 | 16.2% | 16.6% |
|  | Other | 1 | 0.7% | 0.7% |
|  | Not Answered/Not Applicable | 3 | 2.0% |  |
| Total Residents/Fellows | | | | |
|  | <20 | 74 | 50.0% | 51.0% |
|  | 21-50 | 59 | 39.9% | 40.7% |
|  | 51-99 | 12 | 8.1% | 8.3% |
|  | >100 | 3 | 2.0% |  |
| Length of program (years) | | | | |
|  | 2 | 20 | 13.5% | 13.6% |
|  | 3 | 57 | 38.5% | 38.8% |
|  | 4 | 51 | 34.5% | 34.7% |
|  | 5 | 15 | 10.1% | 10.2% |
|  | 6 | 3 | 2.0% | 2.0% |
|  | 7 or more | 1 | 0.7% | 0.7% |
|  | Not Answered/Not Applicable | 1 | 0.7% |  |
| Age |  | | | |
|  | <30 | 6 | 4.1% | 4.1% |
|  | 30-39 | 33 | 22.3% | 22.4% |
|  | 40-49 | 70 | 47.3% | 47.6% |
|  | 50-59 | 24 | 16.2% | 16.3% |
|  | 60-69 | 14 | 9.5% | 9.5% |
|  | Not Answered/Not Applicable | 1 | 0.7% |  |
| Race/Ethnicity | | | | |
|  | American Indian/Alaska Native | 0 | 0.0% | 0.0% |
|  | Asian/Pacific Islander | 21 | 14.2% | 14.8% |
|  | Black/African American | 7 | 4.7% | 4.9% |
|  | Caucasian | 94 | 63.5% | 66.2% |
|  | Latino/Hispanic | 8 | 5.4% | 5.6% |
|  | 2 or more races | 5 | 3.4% | 3.5% |
|  | Other | 7 | 4.7% | 4.9% |
|  | Not Answered/Not Applicable | 6 | 4.1% |  |
| Marital Status | | | | |
|  | Divorced | 8 | 5.4% | 5.6% |
|  | Married | 110 | 74.3% | 77.5% |
|  | Partnered | 6 | 4.1% | 4.2% |
|  | Single | 16 | 10.8% | 11.3% |
|  | Widowed | 2 | 1.4% | 1.4% |
|  | Other | 2 | 1.4% |  |
|  | Not Answered/Not Applicable | 4 | 2.7% |  |
| Have children | | | | |
|  | No | 31 | 20.9% | 21.4% |
|  | Yes | 114 | 77.0% | 78.6% |
|  | Not Answered/Not Applicable | 3 | 2.0% |  |
| If yes, did you have your children while in residency or fellowship? | | | | |
|  | No | 61 | 41.2% | 54.0% |
|  | Yes | 52 | 35.1% | 46.0% |
|  | Not Answered/Not Applicable | 1 | 0.7% |  |
| Live in a state with fertility coverage? | | | | |
|  | No | 101 | 68.2% | 68.7% |
|  | Yes | 46 | 31.1% | 31.3% |
|  | Not Answered/Not Applicable | 1 | 0.7% |  |

| **Table 2. Residency Policies** | | **N** | **Percentage** | **Percentage (excluding Not Answered/Not Applicable)** | |
| --- | --- | --- | --- | --- | --- |
| Amount of maternity leave granted to female students | | | | | |
|  | <2 weeks | 2 | 1.4% | | 1.5% |
|  | 2- <4 weeks | 5 | 3.4% | | 3.7% |
|  | 4 - <6 weeks | 29 | 19.6% | | 21.6% |
|  | 6 - <8 weeks | 48 | 32.4% | | 35.8% |
|  | 8 to <12 weeks | 34 | 23.0% | | 25.4% |
|  | 12+ weeks | 16 | 10.8% | | 11.9% |
|  | Not Answered/Not Applicable | 14 | 9.5% | |  |
| Amount of paternity leave granted to male students | | | | | |
|  | <2 weeks | 43 | 29.1% | | 32.8% |
|  | 2- <4 weeks | 38 | 25.7% | | 29.0% |
|  | 4 - <6 weeks | 16 | 10.8% | | 12.2% |
|  | 6 - <8 weeks | 12 | 8.1% | | 9.2% |
|  | 8 to <12 weeks | 16 | 10.8% | | 12.2% |
|  | 12+ weeks | 6 | 4.1% | | 4.6% |
|  | Not Answered/Not Applicable | 17 | 11.5% | |  |
| During parental leave, how is the majority of missed work covered? (Select all that apply) | | | | | |
|  | By other residents | 121 | 81.8% | | 88.3% |
|  | By extra non-resident help | 43 | 29.1% | | 31.4% |
|  | Resident taking parental leave makes up missed calls | 32 | 21.6% | | 23.4% |
|  | Other | 8 | 5.4% | | 5.8% |
|  | Not Answered/Not Applicable | 11 | 7.4% | |  |
| Are residents allowed to extend maternity leave beyond a set amount (excluding emergency or medically indicated situations)? | | | | | |
|  | Yes | 91 | 61.5% | | 65.9% |
|  | No | 26 | 17.6% | | 18.8% |
|  | Other | 21 | 14.2% | | 15.2% |
|  | Not Answered/Not Applicable | 10 | 6.8% | |  |
| What is the residency insurance (offered through the GME) coverage of infertility treatment at your program? | | | | | |
|  | Covers all aspects of treatment | 11 | 7.4% | | 7.9% |
|  | Covers some aspects of treatment | 28 | 18.9% | | 20.1% |
|  | I don’t know | 83 | 56.1% | | 59.7% |
|  | Not covered | 17 | 11.5% | | 12.2% |
|  | Not Answered/Not Applicable | 9 | 6.1% | |  |
| What is the residency insurance (offered through the GME) coverage of fertility preservation treatment (egg or embryo freezing) at your program? | | | | | |
|  | Covers all aspects of treatment | 2 | 1.4% | | 1.4% |
|  | Covers some aspects of treatment | 5 | 3.4% | | 3.6% |
|  | I don't know | 102 | 68.9% | | 72.9% |
|  | Not covered | 31 | 20.9% | | 22.1% |
|  | Not Answered/Not Applicable | 8 | 5.4% | |  |

| **Table 3. Infertility Support** | | **N** | **Percentage** | | **Percentage (excluding Not Answered/Not Applicable)** | |
| --- | --- | --- | --- | --- | --- | --- |
| How many of your residents have disclosed to you that they are facing infertility or recurrent pregnancy loss (RPL, 2 or more miscarriages)? | | | | | | |
|  | None | 69 | | 46.6% | | 51.9% |
|  | <5% | 40 | | 27.0% | | 30.1% |
|  | 6-10% | 20 | | 13.5% | | 15.0% |
|  | 11-25% | 3 | | 2.0% | | 2.3% |
|  | <25% | 1 | | 0.7% | | 0.8% |
|  | Not Answered/Not Applicable | 15 | | 10.1% | |  |
| What is your estimate of how many residents in your program are facing infertility or recurrent pregnancy loss (RPL, defined as 2 or more miscarriages)? | | | | | | |
|  | None | 40 | 27.0% | | 30.8% | |
|  | <5% | 46 | 31.1% | | 35.4% | |
|  | 6-10% | 37 | 25.0% | | 28.5% | |
|  | 11-25% | 6 | 4.1% | | 4.6% | |
|  | <25% | 1 | 0.7% | | 0.8% | |
|  | Not Answered/Not Applicable | 18 | 12.2% | |  | |
| In your opinion, has the number of residents with fertility issues changed in the past years? | | | | | | |
|  | Decreased | 0 | 0.0% | | 0.0% | |
|  | Increased | 25 | 16.9% | | 42.4% | |
|  | No change | 34 | 23.0% | | 57.6% | |
|  | Not Answered/Not Applicable | 89 | 60.1% | |  | |
| To your knowledge, how many residents in your program have undergone fertility treatment, such as IUI or IVF? | | | | | | |
|  | None | 73 | 49.3% | | 56.6% | |
|  | <5% | 36 | 24.3% | | 27.9% | |
|  | 6-10% | 16 | 10.8% | | 12.4% | |
|  | >11% | 4 | 2.7% | | 3.1% | |
|  | Not Answered/Not Applicable | 19 | 12.8% | |  | |
| What resources exist in your residency program for residents facing infertility/recurrent pregnancy loss? (Check all that apply) | | | | | | |
|  | Time off for appointments | 94 | 63.5% | | 76.4% | |
|  | Moral support from program director | 107 | 72.3% | | 87.0% | |
|  | Trainee discount | 7 | 4.7% | | 5.7% | |
|  | Insurance coverage | 53 | 35.8% | | 43.1% | |
|  | Other financial support | 2 | 1.4% | | 1.6% | |
|  | Other | 15 | 10.1% | | 12.2% | |
|  | Not Answered/Not Applicable | 25 | 16.9% | |  | |
| How supportive do you feel your program is towards residents with fertility issues (infertility or RPL)? | | | | | | |
|  | Very supportive | 59 | 39.9% | | 45.4% | |
|  | Somewhat supportive | 58 | 39.2% | | 44.6% | |
|  | Minimally supportive | 8 | 5.4% | | 6.2% | |
|  | Not supportive | 5 | 3.4% | | 3.8% | |
|  | Not Answered/Not Applicable | 18 | 12.2% | |  | |
| How supportive do you feel personally towards residents with fertility issues (infertility or RPL)? | | | | | | |
|  | Very supportive | 105 | 70.9% | | 80.2% | |
|  | Somewhat supportive | 23 | 15.5% | | 17.6% | |
|  | Minimally supportive | 2 | 1.4% | | 1.5% | |
|  | Not supportive | 1 | 0.7% | | 0.8% | |
|  | Not Answered/Not Applicable | 17 | 11.5% | |  | |
| What is your attitude towards the alignment of your personal level of support and program level of support for residents facing infertility or RPL? | | | | | | |
|  | Aligned in level of support | 79 | 53.4% | | 60.8% | |
|  | Program is less supportive | 41 | 27.7% | | 31.5% | |
|  | Program is more supportive | 2 | 1.4% | | 1.5% | |
|  | Other | 8 | 5.4% | | 6.2% | |
|  | Not Answered/Not Applicable | 18 | 12.2% | |  | |
| Are there measures currently in place to improve support for residents facing infertility or RPL? | | | | | | |
|  | Yes | 7 | 4.7% | | 5.5% | |
|  | No | 109 | 73.6% | | 85.8% | |
|  | Other | 11 | 7.4% | | 8.7% | |
|  | Not Answered/Not Applicable | 21 | 14.2% | |  | |
| Are residents allowed to take time off for treatment for infertility or RPL? | | | | | | |
|  | No | 30 | 20.3% | | 25.2% | |
|  | Yes - 2 days or less per year | 8 | 5.4% | | 6.7% | |
|  | Yes - 3 days to 1 week per year | 29 | 19.6% | | 24.4% | |
|  | Yes - greater than 1 week per year | 52 | 35.1% | | 43.7% | |
|  | Not Answered/Not Applicable | 29 | 19.6% | |  | |
| Do you have any official or standardized policies on taking time off for infertility and RPL treatment? | | | | | | |
|  | Yes | 3 | 2.0% | | 2.3% | |
|  | No - case by case basis | 114 | 77.0% | | 88.4% | |
|  | Other | 12 | 8.1% | | 9.3% | |
|  | Not Answered/Not Applicable | 19 | 12.8% | |  | |
| How many residents have taken time off for infertility or RPL treatment in your program? | | | | | | |
|  | None | 78 | 52.7% | | 60.9% | |
|  | <5% | 36 | 24.3% | | 28.1% | |
|  | 6-10% | 11 | 7.4% | | 8.6% | |
|  | >11% | 3 | 2.0% | | 2.3% | |
|  | Not Answered/Not Applicable | 20 | 13.5% | |  | |

| **Table 4. Fertility Preservation Support** | | **N** | **Percentage** | | **Percentage (excluding Not Answered/Not Applicable)** |
| --- | --- | --- | --- | --- | --- |
| How many of your residents have expressed interest to you in fertility preservation? | | | | | |
|  | None | 97 | | 65.5% | 77.6% |
|  | <5% | 18 | | 12.2% | 14.4% |
|  | 6-10% | 9 | | 6.1% | 7.2% |
|  | >11% | 1 | | 0.7% | 0.8% |
|  | Not Answered/Not Applicable | 23 | | 15.5% |  |
| To your knowledge, how many of your residents have undergone fertility preservation? | | | | | |
|  | None | 106 | | 71.6% | 85.5% |
|  | <5% | 17 | | 11.5% | 13.7% |
|  | >6% | 1 | | 0.7% | 0.8% |
|  | Not Answered/Not Applicable | 24 | | 16.2% |  |
| What resources exist in your residency program for residents interested in fertility preservation? (Check all that apply) | | | | | |
|  | Time off for appointments | 74 | | 50.0% | 66.7% |
|  | Moral support from program director | 92 | | 62.2% | 82.9% |
|  | Trainee discount | 6 | | 4.1% | 5.4% |
|  | Insurance coverage | 24 | | 16.2% | 21.6% |
|  | Other financial support | 1 | | 0.7% | 0.9% |
|  | Other | 13 | | 8.8% | 11.7% |
|  | Not Answered/Not Applicable | 37 | | 25.0% |  |
| How supportive do you feel your program is towards residents interested in fertility preservation? | | | | | |
|  | Very supportive | 48 | | 32.4% | 39.3% |
|  | Somewhat supportive | 59 | | 39.9% | 48.4% |
|  | Minimally supportive | 10 | | 6.8% | 8.2% |
|  | Not supportive | 5 | | 3.4% | 4.1% |
|  | Not Answered/Not Applicable | 26 | | 17.6% |  |
| How supportive do you feel personally towards residents interested in fertility preservation? | | | | | |
|  | Very supportive | 93 | | 62.8% | 73.8% |
|  | Somewhat supportive | 28 | | 18.9% | 22.2% |
|  | Minimally supportive | 5 | | 3.4% | 4.0% |
|  | Not supportive | 0 | | 0.0% | 0.0% |
|  | Not Answered/Not Applicable | 22 | | 14.9% |  |
| What is your attitude towards the alignment of your personal level of support and program level of support for residents interested in fertility preservation? | | | | | |
|  | Aligned in level of support | 75 | | 50.7% | 61.0% |
|  | Program is less supportive | 37 | | 25.0% | 30.1% |
|  | Program is more supportive | 2 | | 1.4% | 1.6% |
|  | Other | 9 | | 6.1% | 7.3% |
|  | Not Answered/Not Applicable | 25 | | 16.9% |  |
| Are there measures currently in place to improve support for residents interested in fertility preservation? | | | | | |
|  | Yes | 7 | | 4.7% | 5.8% |
|  | No | 104 | | 70.3% | 86.7% |
|  | Other | 9 | | 6.1% | 7.5% |
|  | Not Answered/Not Applicable | 28 | | 18.9% |  |
| Are residents allowed to take time off for treatment for fertility preservation? | | | | | |
|  | No | 28 | | 18.9% | 24.6% |
|  | Yes - 2 days or less per year | 13 | | 8.8% | 11.4% |
|  | Yes - 3 days to 1 week per year | 26 | | 17.6% | 22.8% |
|  | Yes - greater than 1 week per year | 47 | | 31.8% | 41.2% |
|  | Not Answered/Not Applicable | 34 | | 23.0% |  |
| Do you have any official or standardized policies on taking time off for fertility preservation? | | | | | |
|  | Yes | 0 | | 0.0% | 0.0% |
|  | No - case by case basis | 111 | | 75.0% | 91.0% |
|  | Other | 11 | | 7.4% | 9.0% |
|  | Not Answered/Not Applicable | 26 | | 17.6% |  |
| How many residents have taken time off for fertility preservation treatment in your program? | | | | | |
|  | None | 109 | | 73.6% | 88.6% |
|  | <5% | 14 | | 9.5% | 11.4% |
|  | >6% | 0 | | 0.0% | 0.0% |
|  | Not Answered/Not Applicable | 25 | | 16.9% |  |

| **Table 5. Fertility and Residency** | | **N** | | **Percentage** | **Percentage (excluding Not Answered/Not Applicable)** |
| --- | --- | --- | --- | --- | --- |
| What is your understanding of  the effect of age on fertility in women? | | | | | |
|  | No effect | 0 | | 0.0% | 0.0% |
|  | Fertility decreases around age 25 | 12 | | 8.1% | 9.6% |
|  | Fertility decreases around age 30 | 55 | | 37.2% | 44.0% |
|  | Fertility decreases around age 35 | 50 | | 33.8% | 40.0% |
|  | Fertility decreases around age 40 | 4 | | 2.7% | 3.2% |
|  | Fertility decreases around age 45 | 0 | | 0.0% | 0.0% |
|  | Other | 4 | | 2.7% | 3.2% |
|  | Not Answered/Not Applicable | 23 | | 15.5% |  |
| What is your understanding of the effect of age on fertility in men? | | | | | |
|  | No effect | | 33 | 22.3% | 26.6% |
|  | Fertility decreases around age 25 | | 0 | 0.0% | 0.0% |
|  | Fertility decreases around age 30 | | 2 | 1.4% | 1.6% |
|  | Fertility decreases around age 35 | | 9 | 6.1% | 7.3% |
|  | Fertility decreases around age 40 | | 38 | 25.7% | 30.6% |
|  | Fertility decreases around age 45 | | 34 | 23.0% | 27.4% |
|  | Other | | 8 | 5.4% | 6.5% |
|  | Not Answered/Not Applicable | | 24 | 16.2% |  |
| How important do you feel it is to increase resources for residents undergoing infertility or RPL treatment? | | | | | |
|  | Very important | | 40 | 27.0% | 32.5% |
|  | Somewhat important | | 54 | 36.5% | 43.9% |
|  | Neither important nor unimportant | | 22 | 14.9% | 17.9% |
|  | Somewhat unimportant | | 4 | 2.7% | 3.3% |
|  | Very unimportant | | 3 | 2.0% | 2.4% |
|  | Not Answered/Not Applicable | | 25 | 16.9% |  |
| How important do you feel it is to increase resources for residents undergoing fertility preservation? | | | | | |
|  | Very important | | 37 | 25.0% | 30.3% |
|  | Somewhat important | | 53 | 35.8% | 43.4% |
|  | Neither important nor unimportant | | 24 | 16.2% | 19.7% |
|  | Somewhat unimportant | | 6 | 4.1% | 4.9% |
|  | Very unimportant | | 2 | 1.4% | 1.6% |
|  | Not Answered/Not Applicable | | 26 | 17.6% |  |
| As a residency director, what is your stance regarding residents trying to get pregnant? | | | | | |
|  | Strongly encourage | | 47 | 31.8% | 38.8% |
|  | Somewhat encourage | | 20 | 13.5% | 16.5% |
|  | Neither encourage nor discourage | | 53 | 35.8% | 43.8% |
|  | Somewhat discourage | | 1 | 0.7% | 0.8% |
|  | Strongly discourage | | 0 | 0.0% | 0.0% |
|  | Not Answered/Not Applicable | | 27 | 18.2% |  |
| Does your opinion on the above differ for male and female residents? | | | | | |
|  | Yes - “discourage” female residents more | | 7 | 4.7% | 5.8% |
|  | Yes - “discourage” male residents more | | 0 | 0.0% | 0.0% |
|  | Yes - other (please specify) | | 0 | 0.0% | 0.0% |
|  | No | | 113 | 76.4% | 93.4% |
|  | Other (please specify) | | 1 | 0.7% | 0.8% |
|  | Not Answered/Not Applicable | | 27 | 18.2% |  |
| A trainee discount would help with the costs of undergoing assisted reproductive technologies in order to conceive. | | | | | |
|  | Strongly agree | | 65 | 43.9% | 52.8% |
|  | Somewhat agree | | 28 | 18.9% | 22.8% |
|  | Neither agree nor disagree | | 11 | 7.4% | 8.9% |
|  | Somewhat disagree | | 3 | 2.0% | 2.4% |
|  | Strongly disagree | | 0 | 0.0% | 0.0% |
|  | No opinion | | 16 | 10.8% | 13.0% |
|  | Not Answered/Not Applicable | | 25 | 16.9% |  |
| A trainee discount would help with the costs of undergoing fertility preservation (egg or embryo freezing). | | | | | |
|  | Strongly agree | | 64 | 43.2% | 52.5% |
|  | Somewhat agree | | 28 | 18.9% | 23.0% |
|  | Neither agree nor disagree | | 10 | 6.8% | 8.2% |
|  | Somewhat disagree | | 1 | 0.7% | 0.8% |
|  | Strongly disagree | | 1 | 0.7% | 0.8% |
|  | No opinion | | 18 | 12.2% | 14.8% |
|  | Not Answered/Not Applicable | | 26 | 17.6% |  |
| In your opinion, what is the biggest barrier to pursuing fertility treatments while in training? | | | | | |
|  | Time | | 52 | 35.1% | 42.3% |
|  | Finances | | 44 | 29.7% | 35.8% |
|  | Lack of information | | 4 | 2.7% | 3.3% |
|  | Lack of partner | | 3 | 2.0% | 2.4% |
|  | Emotional reasons | | 3 | 2.0% | 2.4% |
|  | Other | | 4 | 2.7% | 3.3% |
|  | No opinion | | 13 | 8.8% | 10.6% |
|  | Not Answered/Not Applicable | | 25 | 16.9% |  |
| What do you think should be improved in the current situation in the program regarding support to residents who may be struggling with infertility or interested in fertility preservation? (Check all that apply) | | | | | |
|  | Counseling | | 40 | 27.0% | 33.1% |
|  | Time off for fertility treatment | | 67 | 45.3% | 55.4% |
|  | Expressing personal support during orientation | | 42 | 28.4% | 34.7% |
|  | Financial support | | 59 | 39.9% | 48.8% |
|  | Reach out to department leadership, GME, or dean’s office | | 33 | 22.3% | 27.3% |
|  | Increasing personal awareness of individual needs | | 76 | 51.4% | 62.8% |
|  | Official policies on fertility treatment | | 60 | 40.5% | 49.6% |
|  | Nothing | | 8 | 5.4% | 6.6% |
|  | Other | | 4 | 2.7% | 3.3% |
|  | Not Answered/Not Applicable | | 27 | 18.2% |  |

**Stratified – Male**

N = 145

| **Table 1. Demographics** | | **N** | **Percentage** | **Percentage (excluding Not Answered/Not Applicable)** |
| --- | --- | --- | --- | --- |
| Specialty | | | | |
|  | Allergy and immunology | 1 | 0.7% | 0.7% |
|  | Anesthesiology | 9 | 6.2% | 6.2% |
|  | Dermatology | 6 | 4.1% | 4.1% |
|  | Emergency medicine | 16 | 11.0% | 11.0% |
|  | Endocrinology | 9 | 6.2% | 6.2% |
|  | Family medicine | 13 | 9.0% | 9.0% |
|  | Gastroenterology | 7 | 4.8% | 4.8% |
|  | General surgery | 7 | 4.8% | 4.8% |
|  | Internal medicine | 11 | 7.6% | 7.6% |
|  | Neurological surgery | 1 | 0.7% | 0.7% |
|  | Obstetrics and gynecology | 5 | 3.4% | 3.4% |
|  | Ophthalmology | 8 | 5.5% | 5.5% |
|  | Orthopedic surgery | 6 | 4.1% | 4.1% |
|  | Other (please specify) | 6 | 4.1% | 4.1% |
|  | Other surgical subspecialty (please specify) | 1 | 0.7% | 0.7% |
|  | Otolaryngology | 3 | 2.1% | 2.1% |
|  | Pathology | 1 | 0.7% | 0.7% |
|  | Pediatrics | 2 | 1.4% | 1.4% |
|  | Physical medicine & rehabilitation | 1 | 0.7% | 0.7% |
|  | Plastic surgery | 3 | 2.1% | 2.1% |
|  | Psychiatry | 7 | 4.8% | 4.8% |
|  | Radiology (diagnostic) | 8 | 5.5% | 5.5% |
|  | Thoracic surgery | 4 | 2.8% | 2.8% |
|  | Urology | 10 | 6.9% | 6.9% |
| Region | | | | |
|  | Midwest | 35 | 24.1% | 24.3% |
|  | Northeast | 42 | 29.0% | 29.2% |
|  | South | 44 | 30.3% | 30.6% |
|  | West | 19 | 13.1% | 13.2% |
|  | Other | 4 | 2.8% | 2.8% |
|  | Not Answered/Not Applicable | 1 | 0.7% |  |
| Total Residents/Fellows | | | | |
|  | <20 | 79 | 54.5% | 56.0% |
|  | 21-50 | 46 | 31.7% | 32.6% |
|  | 51-99 | 16 | 11.0% | 11.3% |
|  | >100 | 4 | 2.8% |  |
| Length of program (years) | | | | |
|  | 2 | 17 | 11.7% | 11.7% |
|  | 3 | 65 | 44.8% | 44.8% |
|  | 4 | 30 | 20.7% | 20.7% |
|  | 5 | 26 | 17.9% | 17.9% |
|  | 6 | 4 | 2.8% | 2.8% |
|  | 7 or more | 3 | 2.1% | 2.1% |
| Age |  | | | |
|  | <30 | 0 | 0.0% | 0.0% |
|  | 30-39 | 19 | 13.1% | 13.1% |
|  | 40-49 | 46 | 31.7% | 31.7% |
|  | 50-59 | 43 | 29.7% | 29.7% |
|  | 60-69 | 30 | 20.7% | 20.7% |
|  | 70+ | 7 | 4.8% | 4.8% |
| Race/Ethnicity |  | | | |
|  | American Indian/Alaska Native | 1 | 0.7% | 0.7% |
|  | Asian/Pacific Islander | 14 | 9.7% | 9.7% |
|  | Black/African American | 5 | 3.4% | 3.5% |
|  | Caucasian | 115 | 79.3% | 79.9% |
|  | Latino/Hispanic | 3 | 2.1% | 2.1% |
|  | 2 or more races | 4 | 2.8% | 2.8% |
|  | Other | 2 | 1.4% | 1.4% |
|  | Not Answered/Not Applicable | 1 | 0.7% |  |
| Marital Status |  | | | |
|  | Divorced | 3 | 2.1% | 2.1% |
|  | Married | 128 | 88.3% | 88.9% |
|  | Partnered | 3 | 2.1% | 2.1% |
|  | Single | 10 | 6.9% | 6.9% |
|  | Widowed | 0 | 0.0% | 0.0% |
|  | Other | 0 | 0.0% | 0.0% |
|  | Not Answered/Not Applicable | 1 | 0.7% |  |
| Have children |  |  |  |  |
|  | No | 20 | 13.8% | 14.0% |
|  | Yes | 123 | 84.8% | 86.0% |
|  | Not Answered/Not Applicable | 2 | 1.4% |  |
| If yes, did you have your children while in residency or fellowship? | | | | |
|  | No | 60 | 41.4% | 49.2% |
|  | Yes | 62 | 42.8% | 50.8% |
|  | Not Answered/Not Applicable | 1 | 0.7% |  |
| Live in a state with fertility coverage? | | | | |
|  | No | 100 | 69.0% | 71.4% |
|  | Yes | 40 | 27.6% | 28.6% |
|  | Not Answered/Not Applicable | 5 | 3.4% |  |

| **Table 2. Residency Policies** | | **N** | **Percentage** | **Percentage (excluding Not Answered/Not Applicable)** |
| --- | --- | --- | --- | --- |
| Amount of maternity leave granted to female students | | | | |
|  | <2 weeks | 3 | 2.1% | 2.3% |
|  | 2- <4 weeks | 6 | 4.1% | 4.6% |
|  | 4 - <6 weeks | 31 | 21.4% | 23.8% |
|  | 6 - <8 weeks | 41 | 28.3% | 31.5% |
|  | 8 to <12 weeks | 34 | 23.4% | 26.2% |
|  | 12+ weeks | 15 | 10.3% | 11.5% |
|  | Not Answered/Not Applicable | 15 | 10.3% |  |
| Amount of paternity leave granted to male students | | | | |
|  | <2 weeks | 54 | 37.2% | 41.5% |
|  | 2- <4 weeks | 15 | 10.3% | 11.5% |
|  | 4 - <6 weeks | 12 | 8.3% | 9.2% |
|  | 6 - <8 weeks | 19 | 13.1% | 14.6% |
|  | 8 to <12 weeks | 21 | 14.5% | 16.2% |
|  | 12+ weeks | 9 | 6.2% | 6.9% |
|  | Not Answered/Not Applicable | 15 | 10.3% |  |
| During parental leave, how is the majority of missed work covered? (Select all that apply) | | | | |
|  | By other residents | 121 | 83.4% | 63.7% |
|  | By extra non-resident help | 38 | 26.2% | 20.0% |
|  | Resident taking parental leave makes up missed calls | 26 | 17.9% | 13.7% |
|  | Other | 5 | 3.4% | 2.6% |
|  | Not Answered/Not Applicable | 12 | 8.3% |  |
| Are residents allowed to extend maternity leave beyond a set amount (excluding emergency or medically indicated situations)? | | | | |
|  | Yes | 94 | 64.8% | 70.7% |
|  | No | 22 | 15.2% | 16.5% |
|  | Other | 17 | 11.7% | 12.8% |
|  | Not Answered/Not Applicable | 12 | 8.3% |  |
| What is the residency insurance (offered through the GME) coverage of infertility treatment at your program? | | | | |
|  | Covers all aspects of treatment | 12 | 8.3% | 9.0% |
|  | Covers some aspects of treatment | 15 | 10.3% | 11.3% |
|  | I don’t know | 85 | 58.6% | 63.9% |
|  | Not covered | 21 | 14.5% | 15.8% |
|  | Not Answered/Not Applicable | 12 | 8.3% |  |
| What is the residency insurance (offered through the GME) coverage of fertility preservation treatment (egg or embryo freezing) at your program? | | | | |
|  | Covers all aspects of treatment | 3 | 2.1% | 2.2% |
|  | Covers some aspects of treatment | 3 | 2.1% | 2.2% |
|  | I don't know | 100 | 69.0% | 74.6% |
|  | Not covered | 28 | 19.3% | 20.9% |
|  | Not Answered/Not Applicable | 11 | 7.6% |  |

| **Table 3. Infertility Support** | | **N** | | | **Percentage** | | | | **Percentage (excluding Not Answered/Not Applicable)** | |
| --- | --- | --- | --- | --- | --- | --- | --- | --- | --- | --- |
| How many of your residents have disclosed to you that they are facing infertility or recurrent pregnancy loss (RPL, 2 or more miscarriages)? | | | | | | | | | | |
|  | None | | | 84 | 57.9% | | | | 67.7% | |
|  | <5% | | | 30 | 20.7% | | | | 24.2% | |
|  | 6-10% | | | 6 | 4.1% | | | | 4.8% | |
|  | 11-25% | | | 3 | 2.1% | | | | 2.4% | |
|  | <25% | | | 1 | 0.7% | | | | 0.8% | |
|  | Not Answered/Not Applicable | | | 21 | 14.5% | | | |  | |
| What is your estimate of how many residents in your program are facing infertility or recurrent pregnancy loss (RPL, defined as 2 or more miscarriages)? | | | | | | | | | | |
|  | None | 48 | | | 33.1% | | 39.3% | | | |
|  | <5% | 45 | | | 31.0% | | 36.9% | | | |
|  | 6-10% | 23 | | | 15.9% | | 18.9% | | | |
|  | 11-25% | 5 | | | 3.4% | | 4.1% | | | |
|  | <25% | 1 | | | 0.7% | | 0.8% | | | |
|  | Not Answered/Not Applicable | 23 | | | 15.9% | |  | | | |
| In your opinion, has the number of residents with fertility issues changed in the past years? | | | | | | | |  | | |
|  | Decreased | | 1 | | | 0.7% | | | | 2.2% |
|  | Increased | | 10 | | | 6.9% | | | | 21.7% |
|  | No change | | 35 | | | 24.1% | | | | 76.1% |
|  | Not Answered/Not Applicable | | 99 | | | 68.3% | | | |  |
| To your knowledge, how many residents in your program have undergone fertility treatment, such as IUI or IVF? | | | | | | | | | | |
|  | None | | 82 | | | 56.6% | | | | 66.7% |
|  | <5% | | 29 | | | 20.0% | | | | 23.6% |
|  | 6-10% | | 9 | | | 6.2% | | | | 7.3% |
|  | 11-25% | | 2 | | | 1.4% | | | | 1.6% |
|  | >25% | | 1 | | | 0.7% | | | | 0.8% |
|  | Not Answered/Not Applicable | | 22 | | | 15.2% | | | |  |
| What resources exist in your residency program for residents facing infertility/recurrent pregnancy loss? (Check all that apply) | | | | | | | | | | |
|  | Time off for appointments | | 92 | | | 63.4% | | | | 79.3% |
|  | Moral support from program director | | 96 | | | 66.2% | | | | 82.8% |
|  | Trainee discount | | 5 | | | 3.4% | | | | 4.3% |
|  | Insurance coverage | | 54 | | | 37.2% | | | | 46.6% |
|  | Other financial support | | 1 | | | 0.7% | | | | 0.9% |
|  | Other | | 7 | | | 4.8% | | | | 6.0% |
|  | Not Answered/Not Applicable | | 29 | | | 20.0% | | | |  |
| How supportive do you feel your program is towards residents with fertility issues (infertility or RPL)? | | | | | | | | | | |
|  | Very supportive | | 65 | | | 44.8% | | | | 53.7% |
|  | Somewhat supportive | | 42 | | | 29.0% | | | | 34.7% |
|  | Minimally supportive | | 12 | | | 8.3% | | | | 9.9% |
|  | Not supportive | | 2 | | | 1.4% | | | | 1.7% |
|  | Not Answered/Not Applicable | | 24 | | | 16.6% | | | |  |
| How supportive do you feel personally towards residents with fertility issues (infertility or RPL)? | | | | | | | | | | |
|  | Very supportive | | 99 | | | 68.3% | | | | 79.8% |
|  | Somewhat supportive | | 22 | | | 15.2% | | | | 17.7% |
|  | Minimally supportive | | 3 | | | 2.1% | | | | 2.4% |
|  | Not supportive | | 0 | | | 0.0% | | | | 0.0% |
|  | Not Answered/Not Applicable | | 21 | | | 14.5% | | | |  |
| What is your attitude towards the alignment of your personal level of support and program level of support for residents facing infertility or RPL? | | | | | | | | | | |
|  | Aligned in level of support | | 87 | | | 60.0% | | | | 70.7% |
|  | Program is less supportive | | 26 | | | 17.9% | | | | 21.1% |
|  | Program is more supportive | | 0 | | | 0.0% | | | | 0.0% |
|  | Other | | 10 | | | 6.9% | | | | 8.1% |
|  | Not Answered/Not Applicable | | 22 | | | 15.2% | | | |  |
| Are there measures currently in place to improve support for residents facing infertility or RPL? | | | | | | | | | | |
|  | Yes | | 11 | | | 7.6% | | | | 9.3% |
|  | No | | 95 | | | 65.5% | | | | 80.5% |
|  | Other | | 12 | | | 8.3% | | | | 10.2% |
|  | Not Answered/Not Applicable | | 27 | | | 18.6% | | | |  |
| Are residents allowed to take time off for treatment for infertility or RPL? | | | | | | | | | | |
|  | No | | 15 | | | 10.3% | | | | 13.0% |
|  | Yes - 2 days or less per year | | 12 | | | 8.3% | | | | 10.4% |
|  | Yes - 3 days to 1 week per year | | 29 | | | 20.0% | | | | 25.2% |
|  | Yes - greater than 1 week per year | | 59 | | | 40.7% | | | | 51.3% |
|  | Not Answered/Not Applicable | | 30 | | | 20.7% | | | |  |
| Do you have any official or standardized policies on taking time off for infertility and RPL treatment? | | | | | | | | | | |
|  | Yes | | 3 | | | 2.1% | | | | 2.5% |
|  | No - case by case basis | | 109 | | | 75.2% | | | | 90.8% |
|  | Other | | 8 | | | 5.5% | | | | 6.7% |
|  | Not Answered/Not Applicable | | 25 | | | 17.2% | | | |  |
| How many residents have taken time off for infertility or RPL treatment in your program? | | | | | | | | | | |
|  | None | | 94 | | | 64.8% | | | | 77.7% |
|  | <5% | | 20 | | | 13.8% | | | | 16.5% |
|  | 6-10% | | 6 | | | 4.1% | | | | 5.0% |
|  | >11% | | 1 | | | 0.7% | | | | 0.8% |
|  | Not Answered/Not Applicable | | 24 | | | 16.6% | | | |  |

| **Table 4. Fertility Preservation Support** | | **N** | **Percentage** | **Percentage (excluding Not Answered/Not Applicable)** | |
| --- | --- | --- | --- | --- | --- |
| How many of your residents have expressed interest to you in fertility preservation? | | | | |  |
|  | None | 98 | 67.6% | 83.1% | |
|  | <5% | 15 | 10.3% | 12.7% | |
|  | 6-10% | 4 | 2.8% | 3.4% | |
|  | >11% | 1 | 0.7% | 0.8% | |
|  | Not Answered/Not Applicable | 27 | 18.6% |  | |
| To your knowledge, how many of your residents have undergone fertility preservation? | | | | |  |
|  | None | 104 | 71.7% | 88.1% | |
|  | <5% | 13 | 9.0% | 11.0% | |
|  | >6% | 1 | 0.7% | 0.8% | |
|  | Not Answered/Not Applicable | 27 | 18.6% |  | |
| What resources exist in your residency program for residents interested in fertility preservation? (Check all that apply) | | | | |  |
|  | Time off for appointments | 74 | 51.0% | 69.2% | |
|  | Moral support from program director | 84 | 57.9% | 78.5% | |
|  | Trainee discount | 2 | 1.4% | 1.9% | |
|  | Insurance coverage | 32 | 22.1% | 29.9% | |
|  | Other financial support | 2 | 1.4% | 1.9% | |
|  | Other | 15 | 10.3% | 14.0% | |
|  | Not Answered/Not Applicable | 38 | 26.2% |  | |
| How supportive do you feel your program is towards residents interested in fertility preservation? | | | | |  |
|  | Very supportive | 66 | 45.5% | 57.4% | |
|  | Somewhat supportive | 31 | 21.4% | 27.0% | |
|  | Minimally supportive | 14 | 9.7% | 12.2% | |
|  | Not supportive | 4 | 2.8% | 3.5% | |
|  | Not Answered/Not Applicable | 30 | 20.7% |  | |
| How supportive do you feel personally towards residents interested in fertility preservation? | | | | |  |
|  | Very supportive | 87 | 60.0% | 75.0% | |
|  | Somewhat supportive | 24 | 16.6% | 20.7% | |
|  | Minimally supportive | 4 | 2.8% | 3.4% | |
|  | Not supportive | 1 | 0.7% | 0.9% | |
|  | Not Answered/Not Applicable | 29 | 20.0% |  | |
| What is your attitude towards the alignment of your personal level of support and program level of support for residents interested in fertility preservation? | | | | |  |
|  | Aligned in level of support | 90 | 62.1% | 77.6% | |
|  | Program is less supportive | 19 | 13.1% | 16.4% | |
|  | Program is more supportive | 1 | 0.7% | 0.9% | |
|  | Other | 6 | 4.1% | 5.2% | |
|  | Not Answered/Not Applicable | 29 | 20.0% |  | |
| Are there measures currently in place to improve support for residents interested in fertility preservation? | | | | |  |
|  | Yes | 7 | 4.8% | 6.0% | |
|  | No | 99 | 68.3% | 85.3% | |
|  | Other | 10 | 6.9% | 8.6% | |
|  | Not Answered/Not Applicable | 29 | 20.0% |  | |
| Are residents allowed to take time off for treatment for fertility preservation? | | | | |  |
|  | No | 20 | 13.8% | 17.5% | |
|  | Yes - 2 days or less per year | 18 | 12.4% | 15.8% | |
|  | Yes - 3 days to 1 week per year | 26 | 17.9% | 22.8% | |
|  | Yes - greater than 1 week per year | 50 | 34.5% | 43.9% | |
|  | Not Answered/Not Applicable | 31 | 21.4% |  | |
| Do you have any official or standardized policies on taking time off for fertility preservation? | | | | |  |
|  | Yes | 6 | 4.1% | 5.2% | |
|  | No - case by case basis | 102 | 70.3% | 87.9% | |
|  | Other | 8 | 5.5% | 6.9% | |
|  | Not Answered/Not Applicable | 29 | 20.0% |  | |
| How many residents have taken time off for fertility preservation treatment in your program? | | | | |  |
|  | None | 102 | 70.3% | 87.9% | |
|  | <5% | 12 | 8.3% | 10.3% | |
|  | >6% | 2 | 1.4% | 1.7% | |
|  | Not Answered/Not Applicable | 29 | 20.0% |  | |

| **Table 5. Fertility and Residency** | | | **N** | | | **Percentage** | | | **Percentage (excluding Not Answered/Not Applicable)** | |
| --- | --- | --- | --- | --- | --- | --- | --- | --- | --- | --- |
| What is your understanding of  the effect of age on fertility in women? | | | | | | | | | | |
|  | No effect | 0 | | | 0.0% | | | 0.0% | | |
|  | Fertility decreases around age 25 | 5 | | | 3.4% | | | 4.5% | | |
|  | Fertility decreases around age 30 | 41 | | | 28.3% | | | 36.9% | | |
|  | Fertility decreases around age 35 | 54 | | | 37.2% | | | 48.6% | | |
|  | Fertility decreases around age 40 | 7 | | | 4.8% | | | 6.3% | | |
|  | Fertility decreases around age 45 | 0 | | | 0.0% | | | 0.0% | | |
|  | Other | 4 | | | 2.8% | | | 3.6% | | |
|  | Not Answered/Not Applicable | 34 | | | 23.4% | | |  | | |
| What is your understanding of the effect of age on fertility in men? | | | | | | | | | | |
|  | No effect | | | 31 | | | 21.4% | | | 28.2% |
|  | Fertility decreases around age 25 | | | 0 | | | 0.0% | | | 0.0% |
|  | Fertility decreases around age 30 | | | 4 | | | 2.8% | | | 3.6% |
|  | Fertility decreases around age 35 | | | 5 | | | 3.4% | | | 4.5% |
|  | Fertility decreases around age 40 | | | 34 | | | 23.4% | | | 30.9% |
|  | Fertility decreases around age 45 | | | 30 | | | 20.7% | | | 27.3% |
|  | Other | | | 6 | | | 4.1% | | | 5.5% |
|  | Not Answered/Not Applicable | | | 35 | | | 24.1% | | |  |
| How important do you feel it is to increase resources for residents undergoing infertility or RPL treatment? | | | | | | | | | | |
|  | Very important | | | 18 | | | 12.4% | | | 16.2% |
|  | Somewhat important | | | 53 | | | 36.6% | | | 47.7% |
|  | Neither important nor unimportant | | | 34 | | | 23.4% | | | 30.6% |
|  | Somewhat unimportant | | | 6 | | | 4.1% | | | 5.4% |
|  | Very unimportant | | | 0 | | | 0.0% | | | 0.0% |
|  | Not Answered/Not Applicable | | | 34 | | | 23.4% | | |  |
| How important do you feel it is to increase resources for residents undergoing fertility preservation? | | | | | | | | | | |
|  | Very important | | | 18 | | | 12.4% | | | 16.4% |
|  | Somewhat important | | | 49 | | | 33.8% | | | 44.5% |
|  | Neither important nor unimportant | | | 34 | | | 23.4% | | | 30.9% |
|  | Somewhat unimportant | | | 8 | | | 5.5% | | | 7.3% |
|  | Very unimportant | | | 1 | | | 0.7% | | | 0.9% |
|  | Not Answered/Not Applicable | | | 35 | | | 24.1% | | |  |
| As a residency director, what is your stance regarding residents trying to get pregnant? | | | | | | | | | | |
|  | Strongly encourage | | | 27 | | | 18.6% | | | 24.3% |
|  | Somewhat encourage | | | 7 | | | 4.8% | | | 6.3% |
|  | Neither encourage nor discourage | | | 75 | | | 51.7% | | | 67.6% |
|  | Somewhat discourage | | | 2 | | | 1.4% | | | 1.8% |
|  | Strongly discourage | | | 0 | | | 0.0% | | | 0.0% |
|  | Not Answered/Not Applicable | | | 34 | | | 23.4% | | |  |
| Does your opinion on the above differ for male and female residents? | | | | | | | | | | |
|  | Yes - “discourage” female residents more | | | 1 | | | 0.7% | | | 0.9% |
|  | Yes - “discourage” male residents more | | | 0 | | | 0.0% | | | 0.0% |
|  | Yes - other (please specify) | | | 3 | | | 2.1% | | | 2.7% |
|  | No | | | 107 | | | 73.8% | | | 95.5% |
|  | Other (please specify) | | | 1 | | | 0.7% | | | 0.9% |
|  | Not Answered/Not Applicable | | | 33 | | | 22.8% | | |  |
| A trainee discount would help with the costs of undergoing assisted reproductive technologies in order to conceive. | | | | | | | | | | |
|  | Strongly agree | | | 44 | | | 30.3% | | | 40.4% |
|  | Somewhat agree | | | 28 | | | 19.3% | | | 25.7% |
|  | Neither agree nor disagree | | | 18 | | | 12.4% | | | 16.5% |
|  | Somewhat disagree | | | 1 | | | 0.7% | | | 0.9% |
|  | Strongly disagree | | | 3 | | | 2.1% | | | 2.8% |
|  | No opinion | | | 15 | | | 10.3% | | | 13.8% |
|  | Not Answered/Not Applicable | | | 36 | | | 24.8% | | |  |
| A trainee discount would help with the costs of undergoing fertility preservation (egg or embryo freezing). | | | | | | | | | | |
|  | Strongly agree | | | 48 | | | 33.1% | | | 44.0% |
|  | Somewhat agree | | | 27 | | | 18.6% | | | 24.8% |
|  | Neither agree nor disagree | | | 16 | | | 11.0% | | | 14.7% |
|  | Somewhat disagree | | | 1 | | | 0.7% | | | 0.9% |
|  | Strongly disagree | | | 3 | | | 2.1% | | | 2.8% |
|  | No opinion | | | 14 | | | 9.7% | | | 12.8% |
|  | Not Answered/Not Applicable | | | 36 | | | 24.8% | | |  |
| In your opinion, what is the biggest barrier to pursuing fertility treatments while in training? | | | | | | | | | | |
|  | Time | | | 43 | | | 29.7% | | | 38.7% |
|  | Finances | | | 29 | | | 20.0% | | | 26.1% |
|  | Lack of information | | | 6 | | | 4.1% | | | 5.4% |
|  | Lack of partner | | | 6 | | | 4.1% | | | 5.4% |
|  | Emotional reasons | | | 0 | | | 0.0% | | | 0.0% |
|  | Other | | | 7 | | | 4.8% | | | 6.3% |
|  | No opinion | | | 20 | | | 13.8% | | | 18.0% |
|  | Not Answered/Not Applicable | | | 34 | | | 23.4% | | |  |
| What do you think should be improved in the current situation in the program regarding support to residents who may be struggling with infertility or interested in fertility preservation? (Check all that apply) | | | | | | | | | | |
|  | Counseling | | | 33 | | | 22.8% | | | 30.0% |
|  | Time off for fertility treatment | | | 35 | | | 24.1% | | | 31.8% |
|  | Expressing personal support during orientation | | | 23 | | | 15.9% | | | 20.9% |
|  | Financial support | | | 41 | | | 28.3% | | | 37.3% |
|  | Reach out to department leadership, GME, or dean’s office | | | 20 | | | 13.8% | | | 18.2% |
|  | Increasing personal awareness of individual needs | | | 65 | | | 44.8% | | | 59.1% |
|  | Official policies on fertility treatment | | | 40 | | | 27.6% | | | 36.4% |
|  | Nothing | | | 15 | | | 10.3% | | | 13.6% |
|  | Other | | | 5 | | | 3.4% | | | 4.5% |
|  | Not Answered/Not Applicable | | | 35 | | | 24.1% | | |  |

**Stratified – Non-Surgery**

N = 200

| **Table 1. Demographics** | | **N** | **Percentage** | **Percentage (excluding Not Answered/Not Applicable)** |
| --- | --- | --- | --- | --- |
| Specialty | | | | |
|  | Allergy and immunology | 6 | 3.0% | 3.0% |
|  | Anesthesiology | 15 | 7.5% | 7.5% |
|  | Dermatology | 13 | 6.5% | 6.5% |
|  | Emergency medicine | 33 | 16.5% | 16.5% |
|  | Endocrinology | 19 | 9.5% | 9.5% |
|  | Family medicine | 27 | 13.5% | 13.5% |
|  | Gastroenterology | 10 | 5.0% | 5.0% |
|  | Internal medicine | 20 | 10.0% | 10.0% |
|  | Other (please specify) | 11 | 5.5% | 5.5% |
|  | Pathology | 6 | 3.0% | 3.0% |
|  | Pediatrics | 12 | 6.0% | 6.0% |
|  | Physical medicine & rehabilitation | 4 | 2.0% | 2.0% |
|  | Psychiatry | 10 | 5.0% | 5.0% |
|  | Radiation oncology | 1 | 0.5% | 0.5% |
|  | Radiology (diagnostic) | 13 | 6.5% | 6.5% |
| Region | | | | |
|  | Midwest | 43 | 21.5% | 22.1% |
|  | Northeast | 62 | 31.0% | 31.8% |
|  | South | 56 | 28.0% | 28.7% |
|  | West | 32 | 16.0% | 16.4% |
|  | Other | 2 | 1.0% | 1.0% |
|  | Not Answered/Not Applicable | 5 | 2.5% |  |
| Total Residents/Fellows | | | | |
|  | <20 | 89 | 44.5% | 45.2% |
|  | 21-50 | 77 | 38.5% | 39.1% |
|  | 51-99 | 24 | 12.0% | 12.2% |
|  | >100 | 7 | 3.5% | 3.6% |
|  | Not Answered/Not Applicable | 3 | 1.5% |  |
| Length of program (years) | | | | |
|  | 2 | 35 | 17.5% | 17.8% |
|  | 3 | 108 | 54.0% | 54.8% |
|  | 4 | 51 | 25.5% | 25.9% |
|  | 5 | 3 | 1.5% | 1.5% |
|  | 6 | 0 | 0.0% | 0.0% |
|  | 7 or more | 0 | 0.0% | 0.0% |
|  | Not Answered/Not Applicable | 3 | 1.5% |  |
| Age | | | | |
|  | <30 | 0 | 0.0% | 0.0% |
|  | 30-39 | 32 | 16.0% | 16.3% |
|  | 40-49 | 81 | 40.5% | 41.3% |
|  | 50-59 | 43 | 21.5% | 21.9% |
|  | 60-69 | 34 | 17.0% | 17.3% |
|  | 70+ | 6 | 3.0% | 3.1% |
|  | Not Answered/Not Applicable | 4 | 2.0% |  |
| Gender | | | | |
|  | Female | 98 | 49.0% | 50.0% |
|  | Male | 97 | 48.5% | 49.5% |
|  | Other | 1 | 0.5% | 0.5% |
|  | Not Answered/Not Applicable | 4 | 2.0% |  |
| Race/Ethnicity | | | | |
|  | American Indian/Alaska Native | 1 | 0.5% | 0.5% |
|  | Asian/Pacific Islander | 21 | 10.5% | 11.0% |
|  | Black/African American | 8 | 4.0% | 4.2% |
|  | Caucasian | 140 | 70.0% | 73.3% |
|  | Latino/Hispanic | 8 | 4.0% | 4.2% |
|  | 2 or more races | 7 | 3.5% | 3.7% |
|  | Other | 6 | 3.0% | 3.1% |
|  | Not Answered/Not Applicable | 9 | 4.5% |  |
| Marital Status | | | | |
|  | Divorced | 9 | 4.5% | 4.6% |
|  | Married | 165 | 82.5% | 85.1% |
|  | Partnered | 6 | 3.0% | 3.1% |
|  | Single | 12 | 6.0% | 6.2% |
|  | Widowed | 2 | 1.0% | 1.0% |
|  | Other | 0 | 0.0% | 0.0% |
|  | Not Answered/Not Applicable | 6 | 3.0% |  |
| Have children | | | | |
|  | No | 29 | 14.5% | 14.9% |
|  | Yes | 165 | 82.5% | 85.1% |
|  | Not Answered/Not Applicable | 6 | 3.0% |  |
| If yes, did you have your children while in residency or fellowship? | | | | |
|  | No | 85 | 51.5% | 52.1% |
|  | Yes | 78 | 47.3% | 47.9% |
|  | Not Answered/Not Applicable | 2 | 1.2% |  |
| Live in a state with fertility coverage? | | | | |
|  | No | 133 | 66.5% | 68.9% |
|  | Yes | 60 | 30.0% | 31.1% |
|  | Not Answered/Not Applicable | 7 | 3.5% |  |

| **Table 2. Residency Policies** | | **N** | **Percentage** | **Percentage (excluding Not Answered/Not Applicable)** |
| --- | --- | --- | --- | --- |
| Amount of maternity leave granted to female students | | | | |
|  | <2 weeks | 4 | 2.0% | 2.3% |
|  | 2- <4 weeks | 7 | 3.5% | 4.0% |
|  | 4 - <6 weeks | 32 | 16.0% | 18.1% |
|  | 6 - <8 weeks | 60 | 30.0% | 33.9% |
|  | 8 to <12 weeks | 50 | 25.0% | 28.2% |
|  | 12+ weeks | 24 | 12.0% | 13.6% |
|  | Not Answered/Not Applicable | 23 | 11.5% |  |
| Amount of paternity leave granted to male students | | | | |
|  | <2 weeks | 65 | 32.5% | 37.1% |
|  | 2- <4 weeks | 33 | 16.5% | 18.9% |
|  | 4 - <6 weeks | 19 | 9.5% | 10.9% |
|  | 6 - <8 weeks | 23 | 11.5% | 13.1% |
|  | 8 to <12 weeks | 25 | 12.5% | 14.3% |
|  | 12+ weeks | 10 | 5.0% | 5.7% |
|  | Not Answered/Not Applicable | 25 | 12.5% |  |
| During parental leave, how is the majority of missed work covered? (Select all that apply) | | | | |
|  | By other residents | 155 | 77.5% | 85.6% |
|  | By extra non-resident help | 57 | 28.5% | 31.5% |
|  | Resident taking parental leave makes up missed calls | 37 | 18.5% | 20.4% |
|  | Other | 11 | 5.5% | 6.1% |
|  | Not Answered/Not Applicable | 19 | 9.5% |  |
| Are residents allowed to extend maternity leave beyond a set amount (excluding emergency or medically indicated situations)? | | | | |
|  | Yes | 127 | 63.5% | 69.8% |
|  | No | 29 | 14.5% | 15.9% |
|  | Other | 26 | 13.0% | 14.3% |
|  | Not Answered/Not Applicable | 18 | 9.0% |  |
| What is the residency insurance (offered through the GME) coverage of infertility treatment at your program? | | | | |
|  | Covers all aspects of treatment | 15 | 7.5% | 8.2% |
|  | Covers some aspects of treatment | 25 | 12.5% | 13.7% |
|  | I don’t know | 117 | 58.5% | 63.9% |
|  | Not covered | 26 | 13.0% | 14.2% |
|  | Not Answered/Not Applicable | 17 | 8.5% |  |
| What is the residency insurance (offered through the GME) coverage of fertility preservation treatment (egg or embryo freezing) at your program? | | | | |
|  | Covers all aspects of treatment | 3 | 1.5% | 1.6% |
|  | Covers some aspects of treatment | 4 | 2.0% | 2.2% |
|  | I don't know | 142 | 71.0% | 76.8% |
|  | Not covered | 36 | 18.0% | 19.5% |
|  | Not Answered/Not Applicable | 15 | 7.5% |  |

| **Table 3. Infertility Support** | | **N** | **Percentage** | **Percentage (excluding Not Answered/Not Applicable)** | | |
| --- | --- | --- | --- | --- | --- | --- |
| How many of your residents have disclosed to you that they are facing infertility or recurrent pregnancy loss (RPL, 2 or more miscarriages)? | | | | | | |
|  | None | 98 | 49.0% | | 57.6% | |
|  | <5% | 51 | 25.5% | | 30.0% | |
|  | 6-10% | 16 | 8.0% | | 9.4% | |
|  | 11-25% | 4 | 2.0% | | 2.4% | |
|  | <25% | 1 | 0.5% | | 0.6% | |
|  | Not Answered/Not Applicable | 30 | 15.0% | |  | |
| What is your estimate of how many residents in your program are facing infertility or recurrent pregnancy loss (RPL, defined as 2 or more miscarriages)? | | | | | | |
|  | None | 53 | 26.5% | 31.4% | | |
|  | <5% | 65 | 32.5% | 38.5% | | |
|  | 6-10% | 44 | 22.0% | 26.0% | | |
|  | 11-25% | 6 | 3.0% | 3.6% | | |
|  | <25% | 1 | 0.5% | 0.6% | | |
|  | Not Answered/Not Applicable | 31 | 15.5% |  | | |
| In your opinion, has the number of residents with fertility issues changed in the past years? | | | | | |  |
|  | Decreased | 1 | 0.5% | 1.5% | | |
|  | Increased | 17 | 8.5% | 26.2% | | |
|  | No change | 47 | 23.5% | 72.3% | | |
|  | Not Answered/Not Applicable | 135 | 67.5% |  | | |
| To your knowledge, how many residents in your program have undergone fertility treatment, such as IUI or IVF? | | | | | | |
|  | None | 106 | 53.0% | 63.5% | | |
|  | <5% | 49 | 24.5% | 29.3% | | |
|  | 6-10% | 9 | 4.5% | 5.4% | | |
|  | 11-25% | 3 | 1.5% | 1.8% | | |
|  | >25% | 0 | 0.0% | 0.0% | | |
|  | Not Answered/Not Applicable | 33 | 16.5% |  | | |
| What resources exist in your residency program for residents facing infertility/recurrent pregnancy loss? (Check all that apply) | | | | | | |
|  | Time off for appointments | 120 | 60.0% | 75.9% | | |
|  | Moral support from program director | 136 | 68.0% | 86.1% | | |
|  | Trainee discount | 6 | 3.0% | 3.8% | | |
|  | Insurance coverage | 74 | 37.0% | 46.8% | | |
|  | Other financial support | 1 | 0.5% | 0.6% | | |
|  | Other | 17 | 8.5% | 10.8% | | |
|  | Not Answered/Not Applicable | 42 | 21.0% |  | | |
| How supportive do you feel your program is towards residents with fertility issues (infertility or RPL)? | | | | | | |
|  | Very supportive | 82 | 41.0% | 49.1% | | |
|  | Somewhat supportive | 67 | 33.5% | 40.1% | | |
|  | Minimally supportive | 15 | 7.5% | 9.0% | | |
|  | Not supportive | 3 | 1.5% | 1.8% | | |
|  | Not Answered/Not Applicable | 33 | 16.5% |  | | |
| How supportive do you feel personally towards residents with fertility issues (infertility or RPL)? | | | | | | |
|  | Very supportive | 139 | 69.5% | 82.2% | | |
|  | Somewhat supportive | 26 | 13.0% | 15.4% | | |
|  | Minimally supportive | 4 | 2.0% | 2.4% | | |
|  | Not supportive | 0 | 0.0% | 0.0% | | |
|  | Not Answered/Not Applicable | 31 | 15.5% |  | | |
| What is your attitude towards the alignment of your personal level of support and program level of support for residents facing infertility or RPL? | | | | | | |
|  | Aligned in level of support | 106 | 53.0% | 63.5% | | |
|  | Program is less supportive | 49 | 24.5% | 29.3% | | |
|  | Program is more supportive | 1 | 0.5% | 0.6% | | |
|  | Other | 11 | 5.5% | 6.6% | | |
|  | Not Answered/Not Applicable | 33 |  |  | | |
| Are there measures currently in place to improve support for residents facing infertility or RPL? | | | | | | |
|  | Yes | 9 | 4.5% | 5.6% | | |
|  | No | 138 | 69.0% | 85.7% | | |
|  | Other | 14 | 7.0% | 8.7% | | |
|  | Not Answered/Not Applicable | 39 | 19.5% |  | | |
| Are residents allowed to take time off for treatment for infertility or RPL? | | | | | | |
|  | No | 26 | 13.0% | 16.9% | | |
|  | Yes - 2 days or less per year | 16 | 8.0% | 10.4% | | |
|  | Yes - 3 days to 1 week per year | 38 | 19.0% | 24.7% | | |
|  | Yes - greater than 1 week per year | 74 | 37.0% | 48.1% | | |
|  | Not Answered/Not Applicable | 46 | 23.0% |  | | |
| Do you have any official or standardized policies on taking time off for infertility and RPL treatment? | | | | | | |
|  | Yes | 2 | 1.0% | 1.2% | | |
|  | No - case by case basis | 147 | 73.5% | 89.6% | | |
|  | Other | 15 | 7.5% | 9.1% | | |
|  | Not Answered/Not Applicable | 36 | 18.0% |  | | |
| How many residents have taken time off for infertility or RPL treatment in your program? | | | | | | |
|  | None | 116 | 58.0% | 69.9% | | |
|  | <5% | 40 | 20.0% | 24.1% | | |
|  | 6-10% | 8 | 4.0% | 4.8% | | |
|  | >11% | 2 | 1.0% | 1.2% | | |
|  | Not Answered/Not Applicable | 34 | 17.0% |  | | |

| **Table 4. Fertility Preservation Support** | | **N** | **Percentage** | **Percentage (excluding Not Answered/Not Applicable)** |
| --- | --- | --- | --- | --- |
| How many of your residents have expressed interest to you in fertility preservation? | | | | |
|  | None | 136 | 68.0% | 82.9% |
|  | <5% | 22 | 11.0% | 13.4% |
|  | 6-10% | 6 | 3.0% | 3.7% |
|  | >11% | 0 | 0.0% | 0.0% |
|  | Not Answered/Not Applicable | 36 | 18.0% |  |
| To your knowledge, how many of your residents have undergone fertility preservation? | | | | |
|  | None | 144 | 72.0% | 88.3% |
|  | <5% | 18 | 9.0% | 11.0% |
|  | >6% | 1 | 0.5% | 0.6% |
|  | Not Answered/Not Applicable | 37 | 18.5% |  |
| What resources exist in your residency program for residents interested in fertility preservation? (Check all that apply) | | | | |
|  | Time off for appointments | 105 | 52.5% | 72.4% |
|  | Moral support from program director | 122 | 61.0% | 84.1% |
|  | Trainee discount | 0 | 0.0% | 0.0% |
|  | Insurance coverage | 39 | 19.5% | 26.9% |
|  | Other financial support | 2 | 1.0% | 1.4% |
|  | Other | 18 | 9.0% | 12.4% |
|  | Not Answered/Not Applicable | 55 | 27.5% |  |
| How supportive do you feel your program is towards residents interested in fertility preservation? | | | | |
|  | Very supportive | 76 | 38.0% | 47.2% |
|  | Somewhat supportive | 62 | 31.0% | 38.5% |
|  | Minimally supportive | 15 | 7.5% | 9.3% |
|  | Not supportive | 8 | 4.0% | 5.0% |
|  | Not Answered/Not Applicable | 39 | 19.5% |  |
| How supportive do you feel personally towards residents interested in fertility preservation? | | | | |
|  | Very supportive | 125 | 62.5% | 76.7% |
|  | Somewhat supportive | 30 | 15.0% | 18.4% |
|  | Minimally supportive | 7 | 3.5% | 4.3% |
|  | Not supportive | 1 | 0.5% | 0.6% |
|  | Not Answered/Not Applicable | 37 | 18.5% |  |
| What is your attitude towards the alignment of your personal level of support and program level of support for residents interested in fertility preservation? | | | | |
|  | Aligned in level of support | 110 | 55.0% | 68.3% |
|  | Program is less supportive | 39 | 19.5% | 24.2% |
|  | Program is more supportive | 2 | 1.0% | 1.2% |
|  | Other | 10 | 5.0% | 6.2% |
|  | Not Answered/Not Applicable | 39 | 19.5% |  |
| Are there measures currently in place to improve support for residents interested in fertility preservation? | | | | |
|  | Yes | 6 | 3.0% | 3.8% |
|  | No | 139 | 69.5% | 88.5% |
|  | Other | 12 | 6.0% | 7.6% |
|  | Not Answered/Not Applicable | 43 | 21.5% |  |
| Are residents allowed to take time off for treatment for fertility preservation? | | | | |
|  | No | 31 | 15.5% | 20.0% |
|  | Yes - 2 days or less per year | 25 | 12.5% | 16.1% |
|  | Yes - 3 days to 1 week per year | 34 | 17.0% | 21.9% |
|  | Yes - greater than 1 week per year | 65 | 32.5% | 41.9% |
|  | Not Answered/Not Applicable | 45 | 22.5% |  |
| Do you have any official or standardized policies on taking time off for fertility preservation? | | | | |
|  | Yes | 3 | 1.5% | 1.9% |
|  | No - case by case basis | 142 | 71.0% | 88.8% |
|  | Other | 15 | 7.5% | 9.4% |
|  | Not Answered/Not Applicable | 40 | 20.0% |  |
| How many residents have taken time off for fertility preservation treatment in your program? | | | | |
|  | None | 145 | 72.5% | 90.1% |
|  | <5% | 16 | 8.0% | 9.9% |
|  | >6% | 0 | 0.0% | 0.0% |
|  | Not Answered/Not Applicable | 39 | 19.5% |  |

| **Table 5. Fertility and Residency** | | **N** | **Percentage** | **Percentage (excluding Not Answered/Not Applicable)** |
| --- | --- | --- | --- | --- |
| What is your understanding of  the effect of age on fertility in women? | | | | |
|  | No effect | 0 | 0.0% | 0.0% |
|  | Fertility decreases around age 25 | 12 | 6.0% | 7.6% |
|  | Fertility decreases around age 30 | 61 | 30.5% | 38.9% |
|  | Fertility decreases around age 35 | 70 | 35.0% | 44.6% |
|  | Fertility decreases around age 40 | 9 | 4.5% | 5.7% |
|  | Fertility decreases around age 45 | 0 | 0.0% | 0.0% |
|  | Other | 5 | 2.5% | 3.2% |
|  | Not Answered/Not Applicable | 43 | 21.5% |  |
| What is your understanding of the effect of age on fertility in men? | | | | |
|  | No effect | 47 | 23.5% | 30.3% |
|  | Fertility decreases around age 25 | 0 | 0.0% | 0.0% |
|  | Fertility decreases around age 30 | 4 | 2.0% | 2.6% |
|  | Fertility decreases around age 35 | 7 | 3.5% | 4.5% |
|  | Fertility decreases around age 40 | 46 | 23.0% | 29.7% |
|  | Fertility decreases around age 45 | 41 | 20.5% | 26.5% |
|  | Other | 10 | 5.0% | 6.5% |
|  | Not Answered/Not Applicable | 45 | 22.5% |  |
| How important do you feel it is to increase resources for residents undergoing infertility or RPL treatment? | | | | |
|  | Very important | 35 | 17.5% | 22.4% |
|  | Somewhat important | 72 | 36.0% | 46.2% |
|  | Neither important nor unimportant | 42 | 21.0% | 26.9% |
|  | Somewhat unimportant | 5 | 2.5% | 3.2% |
|  | Very unimportant | 2 | 1.0% | 1.3% |
|  | Not Answered/Not Applicable | 44 | 22.0% |  |
| How important do you feel it is to increase resources for residents undergoing fertility preservation? | | | | |
|  | Very important | 34 | 17.0% | 21.9% |
|  | Somewhat important | 69 | 34.5% | 44.5% |
|  | Neither important nor unimportant | 43 | 21.5% | 27.7% |
|  | Somewhat unimportant | 8 | 4.0% | 5.2% |
|  | Very unimportant | 1 | 0.5% | 0.6% |
|  | Not Answered/Not Applicable | 45 | 22.5% |  |
| As a residency director, what is your stance regarding residents trying to get pregnant? | | | | |
|  | Strongly encourage | 56 | 28.0% | 35.9% |
|  | Somewhat encourage | 18 | 9.0% | 11.5% |
|  | Neither encourage nor discourage | 80 | 40.0% | 51.3% |
|  | Somewhat discourage | 2 | 1.0% | 1.3% |
|  | Strongly discourage | 0 | 0.0% | 0.0% |
|  | Not Answered/Not Applicable | 44 | 22.0% |  |
| Does your opinion on the above differ for male and female residents? | | | | |
|  | Yes - “discourage” female residents more | 5 | 2.5% | 3.2% |
|  | Yes - “discourage” male residents more | 0 | 0.0% | 0.0% |
|  | Yes - other (please specify) | 1 | 0.5% | 0.6% |
|  | No | 149 | 74.5% | 94.9% |
|  | Other (please specify) | 2 | 1.0% | 1.3% |
|  | Not Answered/Not Applicable | 43 | 21.5% |  |
| A trainee discount would help with the costs of undergoing assisted reproductive technologies in order to conceive. | | | | |
|  | Strongly agree | 69 | 34.5% | 44.5% |
|  | Somewhat agree | 42 | 21.0% | 27.1% |
|  | Neither agree nor disagree | 19 | 9.5% | 12.3% |
|  | Somewhat disagree | 3 | 1.5% | 1.9% |
|  | Strongly disagree | 3 | 1.5% | 1.9% |
|  | No opinion | 19 | 9.5% | 12.3% |
|  | Not Answered/Not Applicable | 45 | 22.5% |  |
| A trainee discount would help with the costs of undergoing fertility preservation (egg or embryo freezing). | | | | |
|  | Strongly agree | 71 | 35.5% | 46.1% |
|  | Somewhat agree | 41 | 20.5% | 26.6% |
|  | Neither agree nor disagree | 16 | 8.0% | 10.4% |
|  | Somewhat disagree | 2 | 1.0% | 1.3% |
|  | Strongly disagree | 3 | 1.5% | 1.9% |
|  | No opinion | 21 | 10.5% | 13.6% |
|  | Not Answered/Not Applicable | 46 | 23.0% |  |
| In your opinion, what is the biggest barrier to pursuing fertility treatments while in training? | | | | |
|  | Time | 66 | 33.0% | 42.0% |
|  | Finances | 50 | 25.0% | 31.8% |
|  | Lack of information | 6 | 3.0% | 3.8% |
|  | Lack of partner | 5 | 2.5% | 3.2% |
|  | Emotional reasons | 2 | 1.0% | 1.3% |
|  | Other | 9 | 4.5% | 5.7% |
|  | No opinion | 19 | 9.5% | 12.1% |
|  | Not Answered/Not Applicable | 43 | 21.5% |  |
| What do you think should be improved in the current situation in the program regarding support to residents who may be struggling with infertility or interested in fertility preservation? (Check all that apply) | | | | |
|  | Counseling | 39 | 19.5% | 25.3% |
|  | Time off for fertility treatment | 60 | 30.0% | 39.0% |
|  | Expressing personal support during orientation | 43 | 21.5% | 27.9% |
|  | Financial support | 47 | 23.5% | 30.5% |
|  | Reach out to department leadership, GME, or dean’s office | 37 | 18.5% | 24.0% |
|  | Increasing personal awareness of individual needs | 94 | 47.0% | 61.0% |
|  | Official policies on fertility treatment | 68 | 34.0% | 44.2% |
|  | Nothing | 15 | 7.5% | 9.7% |
|  | Other | 5 | 2.5% | 3.2% |
|  | Not Answered/Not Applicable | 46 | 23.0% |  |

**Stratified – Surgery**

N = 99

| **Table 1. Demographics** | | | **N** | | **Percentage** | | **Percentage (excluding Not Answered/Not Applicable)** |
| --- | --- | --- | --- | --- | --- | --- | --- |
| Specialty | | | | | | | |
|  | General surgery | 16 | | 16.2% | | 16.2% | |
|  | Neurological surgery | 2 | | 2.0% | | 2.0% | |
|  | Obstetrics and gynecology | 29 | | 29.3% | | 29.3% | |
|  | Ophthalmology | 14 | | 14.1% | | 14.1% | |
|  | Orthopedic surgery | 7 | | 7.1% | | 7.1% | |
|  | Other surgical subspecialty (please specify) | 1 | | 1.0% | | 1.0% | |
|  | Otolaryngology | 7 | | 7.1% | | 7.1% | |
|  | Plastic surgery | 3 | | 3.0% | | 3.0% | |
|  | Thoracic surgery | 5 | | 5.1% | | 5.1% | |
|  | Urology | 15 | | 15.2% | | 15.2% | |
| Region | | | | | | | |
|  | Midwest | 22 | | 22.2% | | 22.4% | |
|  | Northeast | 34 | | 34.3% | | 34.7% | |
|  | South | 27 | | 27.3% | | 27.6% | |
|  | West | 12 | | 12.1% | | 12.2% | |
|  | Other | 3 | | 3.0% | | 3.1% | |
|  | Not Answered/Not Applicable | 1 | | 1.0% | |  | |
| Total Residents/Fellows | | | | | | | |
|  | <20 | 68 | | 68.7% | | 68.7% | |
|  | 21-50 | 28 | | 28.3% | | 28.3% | |
|  | 51-99 | 3 | | 3.0% | | 3.0% | |
|  | >100 | 0 | | 0.0% | | 0.0% | |
| Length of program (years) | | | | | | | |
|  | 2 | 4 | | 4.0% | | 4.0% | |
|  | 3 | 14 | | 14.1% | | 14.1% | |
|  | 4 | 31 | | 31.3% | | 31.3% | |
|  | 5 | 39 | | 39.4% | | 39.4% | |
|  | 6 | 7 | | 7.1% | | 7.1% | |
|  | 7 or more | 4 | | 4.0% | | 4.0% | |
| Age |  | | | | | | |
|  | <30 | 6 | | 6.1% | | 6.1% | |
|  | 30-39 | 20 | | 20.2% | | 20.2% | |
|  | 40-49 | 36 | | 36.4% | | 36.4% | |
|  | 50-59 | 24 | | 24.2% | | 24.2% | |
|  | 60-69 | 12 | | 12.1% | | 12.1% | |
|  | 70+ | 1 | | 1.0% | | 1.0% | |
| Gender | | | | | | | |
|  | Female | 49 | | 49.5% | | 50.0% | |
|  | Male | 48 | | 48.5% | | 49.0% | |
|  | Other | 1 | | 1.0% | | 1.0% | |
|  | Not Answered/Not Applicable | 1 | | 1.0% | |  | |
| Race/Ethnicity | | | | | | | |
|  | American Indian/Alaska Native | 0 | | 0.0% | | 0.0% | |
|  | Asian/Pacific Islander | 14 | | 14.1% | | 14.4% | |
|  | Black/African American | 4 | | 4.0% | | 4.1% | |
|  | Caucasian | 69 | | 69.7% | | 71.1% | |
|  | Latino/Hispanic | 4 | | 4.0% | | 4.1% | |
|  | 2 or more races | 2 | | 2.0% | | 2.1% | |
|  | Other | 4 | | 4.0% | | 4.1% | |
|  | Not Answered/Not Applicable | 2 | | 2.0% | |  | |
| Marital Status | | | | | | | |
|  | Divorced | 2 | | 2.0% | | 2.1% | |
|  | Married | 75 | | 75.8% | | 77.3% | |
|  | Partnered | 4 | | 4.0% | | 4.1% | |
|  | Single | 14 | | 14.1% | | 14.4% | |
|  | Widowed | 0 | | 0.0% | | 0.0% | |
|  | Other | 2 | | 2.0% | | 2.1% | |
|  | Not Answered/Not Applicable | 1 | | 1.0% | |  | |
| Have children | | | | | | | |
|  | No | 22 | | 22.2% | | 22.7% | |
|  | Yes | 75 | | 75.8% | | 77.3% | |
|  | Not Answered/Not Applicable | 2 | | 2.0% | |  | |
| If yes, did you have your children while in residency or fellowship? | | | | | | | |
|  | No | 38 | | 50.7% | | 50.7% | |
|  | Yes | 37 | | 49.3% | | 49.3% | |
| Live in a state with fertility coverage? | | | | | | | |
|  | No | 71 | | 71.7% | | 73.2% | |
|  | Yes | 26 | | 26.3% | | 26.8% | |
|  | Not Answered/Not Applicable | 2 | | 2.0% | |  | |

| **Table 2. Residency Policies** | | **N** | **Percentage** | | | **Percentage (excluding Not Answered/Not Applicable)** | |
| --- | --- | --- | --- | --- | --- | --- | --- |
| Amount of maternity leave granted to female students | | | | | | | |
|  | <2 weeks | 1 | 1.0% | | | 1.1% | |
|  | 2- <4 weeks | 4 | 4.0% | | | 4.5% | |
|  | 4 - <6 weeks | 28 | 28.3% | | | 31.8% | |
|  | 6 - <8 weeks | 31 | 31.3% | | | 35.2% | |
|  | 8 to <12 weeks | 17 | 17.2% | | | 19.3% | |
|  | 12+ weeks | 7 | 7.1% | | | 8.0% | |
|  | Not Answered/Not Applicable | 11 | 11.1% | | |  | |
| Amount of paternity leave granted to male students | | | | | | | |
|  | <2 weeks | 34 | 34.3% | | | 39.1% | |
|  | 2- <4 weeks | 20 | 20.2% | | | 23.0% | |
|  | 4 - <6 weeks | 9 | 9.1% | | | 10.3% | |
|  | 6 - <8 weeks | 8 | 8.1% | | | 9.2% | |
|  | 8 to <12 weeks | 11 | 11.1% | | | 12.6% | |
|  | 12+ weeks | 5 | 5.1% | | | 5.7% | |
|  | Not Answered/Not Applicable | 12 |  | | |  | |
| During parental leave, how is the majority of missed work covered? (Select all that apply) | | | | | | | |
|  | By other residents | 88 | | 88.9% | | 97.8% | |
|  | By extra non-resident help | 23 | | 23.2% | | 25.6% | |
|  | Resident taking parental leave makes up missed calls | 21 | | 21.2% | | 23.3% | |
|  | Other | 2 | | 2.0% | | 2.2% | |
|  | Not Answered/Not Applicable | 9 | | 9.1% | |  | |
| Are residents allowed to extend maternity leave beyond a set amount (excluding emergency or medically indicated situations)? | | | | | | | |
|  | Yes | 59 | | | 59.6% | | 65.6% |
|  | No | 19 | | | 19.2% | | 21.1% |
|  | Other | 12 | | | 12.1% | | 13.3% |
|  | Not Answered/Not Applicable | 9 | | | 9.1% | |  |
| What is the residency insurance (offered through the GME) coverage of infertility treatment at your program? | | | | | | | |
|  | Covers all aspects of treatment | 8 | | | 8.1% | | 8.9% |
|  | Covers some aspects of treatment | 17 | | | 17.2% | | 18.9% |
|  | I don’t know | 53 | | | 53.5% | | 58.9% |
|  | Not covered | 12 | | | 12.1% | | 13.3% |
|  | Not Answered/Not Applicable | 9 | | | 9.1% | |  |
| What is the residency insurance (offered through the GME) coverage of fertility preservation treatment (egg or embryo freezing) at your program? | | | | | | | |
|  | Covers all aspects of treatment | 2 | | | 2.0% | | 2.2% |
|  | Covers some aspects of treatment | 3 | | | 3.0% | | 3.3% |
|  | I don't know | 62 | | | 62.6% | | 68.9% |
|  | Not covered | 23 | | | 23.2% | | 25.6% |
|  | Not Answered/Not Applicable | 9 | | | 9.1% | |  |

| **Table 3. Infertility Support** | | **N** | **Percentage** | **Percentage (excluding Not Answered/Not Applicable)** |
| --- | --- | --- | --- | --- |
| How many of your residents have disclosed to you that they are facing infertility or recurrent pregnancy loss (RPL, 2 or more miscarriages)? | | | | |
|  | None | 57 | 57.6% | 64.8% |
|  | <5% | 18 | 18.2% | 20.5% |
|  | 6-10% | 10 | 10.1% | 11.4% |
|  | 11-25% | 2 | 2.0% | 2.3% |
|  | <25% | 1 | 1.0% | 1.1% |
|  | Not Answered/Not Applicable | 11 | 11.1% |  |
| What is your estimate of how many residents in your program are facing infertility or recurrent pregnancy loss (RPL, defined as 2 or more miscarriages)? | | | | |
|  | None | 36 | 36.4% | 42.4% |
|  | <5% | 27 | 27.3% | 31.8% |
|  | 6-10% | 16 | 16.2% | 18.8% |
|  | 11-25% | 5 | 5.1% | 5.9% |
|  | <25% | 1 | 1.0% | 1.2% |
|  | Not Answered/Not Applicable | 14 | 14.1% |  |
| In your opinion, has the number of residents with fertility issues changed in the past years? | | | |  |
|  | Decreased | 0 | 0.0% | 0.0% |
|  | Increased | 18 | 18.2% | 45.0% |
|  | No change | 22 | 22.2% | 55.0% |
|  | Not Answered/Not Applicable | 59 | 59.6% |  |
| To your knowledge, how many residents in your program have undergone fertility treatment, such as IUI or IVF? | | | | |
|  | None | 51 | 51.5% | 59.3% |
|  | <5% | 15 | 15.2% | 17.4% |
|  | 6-10% | 16 | 16.2% | 18.6% |
|  | 11-25% | 3 | 3.0% | 3.5% |
|  | >25% | 1 | 1.0% | 1.2% |
|  | Not Answered/Not Applicable | 13 | 13.1% |  |
| What resources exist in your residency program for residents facing infertility/recurrent pregnancy loss? (Check all that apply) | | | | |
|  | Time off for appointments | 65 | 65.7% | 79.3% |
|  | Moral support from program director | 65 | 65.7% | 79.3% |
|  | Trainee discount | 6 | 6.1% | 7.3% |
|  | Insurance coverage | 33 | 33.3% | 40.2% |
|  | Other financial support | 2 | 2.0% | 2.4% |
|  | Other | 8 | 8.1% | 9.8% |
|  | Not Answered/Not Applicable | 17 | 17.2% |  |
| How supportive do you feel your program is towards residents with fertility issues (infertility or RPL)? | | | | |
|  | Very supportive | 41 | 41.4% | 48.2% |
|  | Somewhat supportive | 34 | 34.3% | 40.0% |
|  | Minimally supportive | 6 | 6.1% | 7.1% |
|  | Not supportive | 4 | 4.0% | 4.7% |
|  | Not Answered/Not Applicable | 14 | 14.1% |  |
| How supportive do you feel personally towards residents with fertility issues (infertility or RPL)? | | | | |
|  | Very supportive | 65 | 65.7% | 74.7% |
|  | Somewhat supportive | 20 | 20.2% | 23.0% |
|  | Minimally supportive | 1 | 1.0% | 1.1% |
|  | Not supportive | 1 | 1.0% | 1.1% |
|  | Not Answered/Not Applicable | 12 | 12.1% |  |
| What is your attitude towards the alignment of your personal level of support and program level of support for residents facing infertility or RPL? | | | | |
|  | Aligned in level of support | 60 | 60.6% | 69.0% |
|  | Program is less supportive | 19 | 19.2% | 21.8% |
|  | Program is more supportive | 1 | 1.0% | 1.1% |
|  | Other | 7 | 7.1% | 8.0% |
|  | Not Answered/Not Applicable | 12 | 12.1% |  |
| Are there measures currently in place to improve support for residents facing infertility or RPL? | | | | |
|  | Yes | 9 | 9.1% | 10.5% |
|  | No | 68 | 68.7% | 79.1% |
|  | Other | 9 | 9.1% | 10.5% |
|  | Not Answered/Not Applicable | 13 | 13.1% |  |
| Are residents allowed to take time off for treatment for infertility or RPL? | | |  |  |
|  | No | 19 | 19.2% | 23.2% |
|  | Yes - 2 days or less per year | 5 | 5.1% | 6.1% |
|  | Yes - 3 days to 1 week per year | 20 | 20.2% | 24.4% |
|  | Yes - greater than 1 week per year | 38 | 38.4% | 46.3% |
|  | Not Answered/Not Applicable | 17 | 17.2% |  |
| Do you have any official or standardized policies on taking time off for infertility and RPL treatment? | | | | |
|  | Yes | 4 | 4.0% | 4.7% |
|  | No - case by case basis | 77 | 77.8% | 89.5% |
|  | Other | 5 | 5.1% | 5.8% |
|  | Not Answered/Not Applicable | 13 | 13.1% |  |
| How many residents have taken time off for infertility or RPL treatment in your program? | | | | |
|  | None | 58 | 58.6% | 69.0% |
|  | <5% | 15 | 15.2% | 17.9% |
|  | 6-10% | 9 | 9.1% | 10.7% |
|  | >11% | 2 | 2.0% | 2.4% |
|  | Not Answered/Not Applicable | 15 | 15.2% |  |

| **Table 4. Fertility Preservation Support** | | **N** | **Percentage** | **Percentage (excluding Not Answered/Not Applicable)** |
| --- | --- | --- | --- | --- |
| How many of your residents have expressed interest to you in fertility preservation? | | | | |
|  | None | 61 | 61.6% | 75.3% |
|  | <5% | 11 | 11.1% | 13.6% |
|  | 6-10% | 7 | 7.1% | 8.6% |
|  | >11% | 2 | 2.0% | 2.5% |
|  | Not Answered/Not Applicable | 18 | 18.2% |  |
| To your knowledge, how many of your residents have undergone fertility preservation? | | | | |
|  | None | 68 | 68.7% | 84.0% |
|  | <5% | 12 | 12.1% | 14.8% |
|  | >6% | 1 | 1.0% | 1.2% |
|  | Not Answered/Not Applicable | 18 | 18.2% |  |
| What resources exist in your residency program for residents interested in fertility preservation? (Check all that apply) | | | | |
|  | Time off for appointments | 54 | 54.5% | 72.0% |
|  | Moral support from program director | 55 | 55.6% | 73.3% |
|  | Trainee discount | 5 | 5.1% | 6.7% |
|  | Insurance coverage | 17 | 17.2% | 22.7% |
|  | Other financial support | 1 | 1.0% | 1.3% |
|  | Other | 10 | 10.1% | 13.3% |
|  | Not Answered/Not Applicable | 24 | 24.2% |  |
| How supportive do you feel your program is towards residents interested in fertility preservation? | | | | |
|  | Very supportive | 38 | 38.4% | 48.7% |
|  | Somewhat supportive | 29 | 29.3% | 37.2% |
|  | Minimally supportive | 10 | 10.1% | 12.8% |
|  | Not supportive | 1 | 1.0% | 1.3% |
|  | Not Answered/Not Applicable | 21 | 21.2% |  |
| How supportive do you feel personally towards residents interested in fertility preservation? | | | | |
|  | Very supportive | 56 | 56.6% | 69.1% |
|  | Somewhat supportive | 23 | 23.2% | 28.4% |
|  | Minimally supportive | 2 | 2.0% | 2.5% |
|  | Not supportive | 0 | 0.0% | 0.0% |
|  | Not Answered/Not Applicable | 18 | 18.2% |  |
| What is your attitude towards the alignment of your personal level of support and program level of support for residents interested in fertility preservation? | | | | |
|  | Aligned in level of support | 56 | 56.6% | 70.0% |
|  | Program is less supportive | 18 | 18.2% | 22.5% |
|  | Program is more supportive | 1 | 1.0% | 1.3% |
|  | Other | 5 | 5.1% | 6.3% |
|  | Not Answered/Not Applicable | 19 | 19.2% |  |
| Are there measures currently in place to improve support for residents interested in fertility preservation? | | | | |
|  | Yes | 8 | 8.1% | 9.9% |
|  | No | 66 | 66.7% | 81.5% |
|  | Other | 7 | 7.1% | 8.6% |
|  | Not Answered/Not Applicable | 18 | 18.2% |  |
| Are residents allowed to take time off for treatment for fertility preservation? | | | | |
|  | No | 17 | 17.2% | 22.7% |
|  | Yes - 2 days or less per year | 7 | 7.1% | 9.3% |
|  | Yes - 3 days to 1 week per year | 19 | 19.2% | 25.3% |
|  | Yes - greater than 1 week per year | 32 | 32.3% | 42.7% |
|  | Not Answered/Not Applicable | 24 | 24.2% |  |
| Do you have any official or standardized policies on taking time off for fertility preservation? | | | | |
|  | Yes | 3 | 3.0% | 3.8% |
|  | No - case by case basis | 73 | 73.7% | 91.3% |
|  | Other | 4 | 4.0% | 5.0% |
|  | Not Answered/Not Applicable | 19 | 19.2% |  |
| How many residents have taken time off for fertility preservation treatment in your program? | | | | |
|  | None | 68 | 68.7% | 85.0% |
|  | <5% | 10 | 10.1% | 12.5% |
|  | >6% | 2 | 2.0% | 2.5% |
|  | Not Answered/Not Applicable | 19 | 19.2% |  |

| **Table 5. Fertility and Residency** | | **N** | **Percentage** | **Percentage (excluding Not Answered/Not Applicable)** |
| --- | --- | --- | --- | --- |
| What is your understanding of  the effect of age on fertility in women? | | | | |
|  | No effect | 0 | 0.0% | 0.0% |
|  | Fertility decreases around age 25 | 5 | 5.1% | 6.2% |
|  | Fertility decreases around age 30 | 36 | 36.4% | 44.4% |
|  | Fertility decreases around age 35 | 35 | 35.4% | 43.2% |
|  | Fertility decreases around age 40 | 2 | 2.0% | 2.5% |
|  | Fertility decreases around age 45 | 0 | 0.0% | 0.0% |
|  | Other | 3 | 3.0% | 3.7% |
|  | Not Answered/Not Applicable | 18 | 18.2% |  |
| What is your understanding of the effect of age on fertility in men? | | | | |
|  | No effect | 18 | 18.2% | 22.2% |
|  | Fertility decreases around age 25 | 0 | 0.0% | 0.0% |
|  | Fertility decreases around age 30 | 2 | 2.0% | 2.5% |
|  | Fertility decreases around age 35 | 7 | 7.1% | 8.6% |
|  | Fertility decreases around age 40 | 27 | 27.3% | 33.3% |
|  | Fertility decreases around age 45 | 23 | 23.2% | 28.4% |
|  | Other | 4 | 4.0% | 4.9% |
|  | Not Answered/Not Applicable | 18 | 18.2% |  |
| How important do you feel it is to increase resources for residents undergoing infertility or RPL treatment? | | | | |
|  | Very important | 24 | 24.2% | 30.0% |
|  | Somewhat important | 35 | 35.4% | 43.8% |
|  | Neither important nor unimportant | 15 | 15.2% | 18.8% |
|  | Somewhat unimportant | 5 | 5.1% | 6.3% |
|  | Very unimportant | 1 | 1.0% | 1.3% |
|  | Not Answered/Not Applicable | 19 | 19.2% |  |
| How important do you feel it is to increase resources for residents undergoing fertility preservation? | | | | |
|  | Very important | 22 | 22.2% | 27.8% |
|  | Somewhat important | 33 | 33.3% | 41.8% |
|  | Neither important nor unimportant | 16 | 16.2% | 20.3% |
|  | Somewhat unimportant | 6 | 6.1% | 7.6% |
|  | Very unimportant | 2 | 2.0% | 2.5% |
|  | Not Answered/Not Applicable | 20 | 20.2% |  |
| As a residency director, what is your stance regarding residents trying to get pregnant? | | | | |
|  | Strongly encourage | 19 | 19.2% | 24.4% |
|  | Somewhat encourage | 9 | 9.1% | 11.5% |
|  | Neither encourage nor discourage | 49 | 49.5% | 62.8% |
|  | Somewhat discourage | 1 | 1.0% | 1.3% |
|  | Strongly discourage | 0 | 0.0% | 0.0% |
|  | Not Answered/Not Applicable | 21 | 21.2% |  |
| Does your opinion on the above differ for male and female residents? | | | | |
|  | Yes - “discourage” female residents more | 3 | 3.0% | 3.8% |
|  | Yes - “discourage” male residents more | 0 | 0.0% | 0.0% |
|  | Yes - other (please specify) | 2 | 2.0% | 2.6% |
|  | No | 73 | 73.7% | 93.6% |
|  | Other (please specify) | 0 | 0.0% | 0.0% |
|  | Not Answered/Not Applicable | 21 | 21.2% |  |
| A trainee discount would help with the costs of undergoing assisted reproductive technologies in order to conceive. | | | | |
|  | Strongly agree | 41 | 41.4% | 51.9% |
|  | Somewhat agree | 14 | 14.1% | 17.7% |
|  | Neither agree nor disagree | 10 | 10.1% | 12.7% |
|  | Somewhat disagree | 1 | 1.0% | 1.3% |
|  | Strongly disagree | 0 | 0.0% | 0.0% |
|  | No opinion | 13 | 13.1% | 16.5% |
|  | Not Answered/Not Applicable | 20 | 20.2% |  |
| A trainee discount would help with the costs of undergoing fertility preservation (egg or embryo freezing). | | | | |
|  | Strongly agree | 42 | 42.4% | 53.2% |
|  | Somewhat agree | 14 | 14.1% | 17.7% |
|  | Neither agree nor disagree | 10 | 10.1% | 12.7% |
|  | Somewhat disagree | 0 | 0.0% | 0.0% |
|  | Strongly disagree | 1 | 1.0% | 1.3% |
|  | No opinion | 12 | 12.1% | 15.2% |
|  | Not Answered/Not Applicable | 20 | 20.2% |  |
| In your opinion, what is the biggest barrier to pursuing fertility treatments while in training? | | | | |
|  | Time | 30 | 30.3% | 38.0% |
|  | Finances | 24 | 24.2% | 30.4% |
|  | Lack of information | 4 | 4.0% | 5.1% |
|  | Lack of partner | 4 | 4.0% | 5.1% |
|  | Emotional reasons | 1 | 1.0% | 1.3% |
|  | Other | 2 | 2.0% | 2.5% |
|  | No opinion | 14 | 14.1% | 17.7% |
|  | Not Answered/Not Applicable | 20 | 20.2% |  |
| What do you think should be improved in the current situation in the program regarding support to residents who may be struggling with infertility or interested in fertility preservation? (Check all that apply) | | | | |
|  | Counseling | 32 | 32.3% | 40.5% |
|  | Time off for fertility treatment | 32 | 32.3% | 40.5% |
|  | Expressing personal support during orientation | 21 | 21.2% | 26.6% |
|  | Financial support | 32 | 32.3% | 40.5% |
|  | Reach out to department leadership, GME, or dean’s office | 17 | 17.2% | 21.5% |
|  | Increasing personal awareness of individual needs | 48 | 48.5% | 60.8% |
|  | Official policies on fertility treatment | 34 | 34.3% | 43.0% |
|  | Nothing | 8 | 8.1% | 10.1% |
|  | Other | 4 | 4.0% | 5.1% |
|  | Not Answered/Not Applicable | 20 | 20.2% |  |
