# Supplementary material for: Fertility Preservation and Infertility Treatment in Medical Training: An Assessment of Residency and Fellowship Program Directors' Attitudes
Source: Womens Health Rep (New Rochelle). 2021 Dec 7;2(1):576–85. doi: 10.1089/whr.2021.0044 (PMC8820404; doi:10.1089/whr.2021.0044)
Supplement: Supplemental data [file Suppl_Appendix_TableSA3.docx]

**APPENDIX C – Stata Multivariate Logarithmic Regression Output**

**Program Support for Infertility Treatment**

|  | Odds Ratio | 95% CI | P-Value |
| --- | --- | --- | --- |
| Specialty | 0.85 | (0.31-2.32) | 0.74 |
| Region | 0.96 | (0.61-1.52) | 0.87 |
| State mandate | 0.85 | (0.30-2.44) | 0.76 |
| Age | 2.52 | (0.81-7.84) | 0.81 |
| Gender | 0.71 | (0.26-1.96) | 0.51 |
| Marital status | 1 (omitted) |  |  |
| Have children | 1 (omitted) |  |  |
| Children during residency | 0.85 | (0.32-2.24) | 0.32 |

**Personal Support for Infertility Treatment**

|  | Odds Ratio | 95% CI | P-Value |
| --- | --- | --- | --- |
| Specialty | 1.06 | (0.08-13.65) | 0.97 |
| Region | 0.37 | (0.08-1.66) | 0.20 |
| State mandate | 1 (omitted) |  |  |
| Age | 2.35 | (0.17-33.31) | 0.53 |
| Gender | 0.40 | (0.03-6.14) | 0.51 |
| Marital status | 1 (omitted) |  |  |
| Have children | 1 (omitted) |  |  |
| Children during residency | 3.30 | (0.25-42.98) | 0.51 |

**Program Support for Fertility Preservation**

|  | Odds Ratio | 95% CI | P-Value |
| --- | --- | --- | --- |
| Specialty | 0.88 | (0.34-2.24) | 0.79 |
| Region | 1.00 | (0.66-1.54) | 0.87 |
| State mandate | 0.57 | (0.22-1.45) | 0.24 |
| Age | 2.04 | (0.78-5.36) | 0.15 |
| Gender | 0.44 | (0.17-1.11) | 0.08 |
| Marital status | 0.73 | (0.09-6.18) | 0.78 |
| Have children | 1 (omitted) |  |  |
| Children during residency | 1.06 | (0.44-2.58) | 0.90 |

**Personal Support for Fertility Preservation**

|  | Odds Ratio | 95% CI | P-Value |
| --- | --- | --- | --- |
| Specialty | 2.90 | (0.33-25.3) | 0.33 |
| Region | 1.02 | (0.47-2.20) | 0.96 |
| State mandate | 0.58 | (0.12-2.90) | 0.51 |
| Age | 5.58 | (0.62-45.96) | 0.12 |
| Gender | 0.49 | (0.10-2.40) | 0.38 |
| Marital status | 1 (omitted) |  |  |
| Have children | 1 (omitted) |  |  |
| Children during residency | 1.09 | (0.22-5.28) | 0.92 |
